# Three hundred years of Palmyrene history. Unlocking archaeological data for studying past societal transformations

**DOI:** 10.1371/journal.pone.0256081

**Published:** 2021-11-03

**Authors:** Rubina Raja, Olympia Bobou, Iza Romanowska

**Affiliations:** 1 Centre for Urban Network Evolutions, Aarhus University, Højbjerg, Denmark; 2 Department of Classical Studies, School of Culture and Society, Aarhus University, Aarhus, Denmark; 3 Aarhus Institute of Advanced Studies, Aarhus University, Aarhus, Denmark; University of Michigan, UNITED STATES

## Abstract

While archaeological sciences have made great advances over the last decades through combining archaeological evidence and natural sciences in order to push borders for the understanding of archaeological contexts, traditional archaeology still holds an immense latent potential. Such potential can be realized through baseline projects that pull together unexplored bodies of material culture and study these in detail in order to investigate their significance for the understanding of the human past. This paper presents such a large-scale baseline study and focuses on the presentation of the results emerging from the recently compiled corpus of more than 3700 funerary portraits stemming from one location in the ancient world, Roman Palmyra, an oasis city in the Syrian Desert. The analysis of the chronological development of the numerous portraits allows us to follow the fluctuations in the production of these portraits over approximately 300 years. Here we discuss and review the developments in connection with historical sources and discuss until now unknown events, which have emerged through the data analysis. The paper brings to the forefront the significance of social science baseline projects, which often do not receive enough attention or funding, but which in fact are fundamental for furthering our understanding of the human past and push borders for the directions in which we can take such studies in the future.

## Introduction

**Fig 1:**
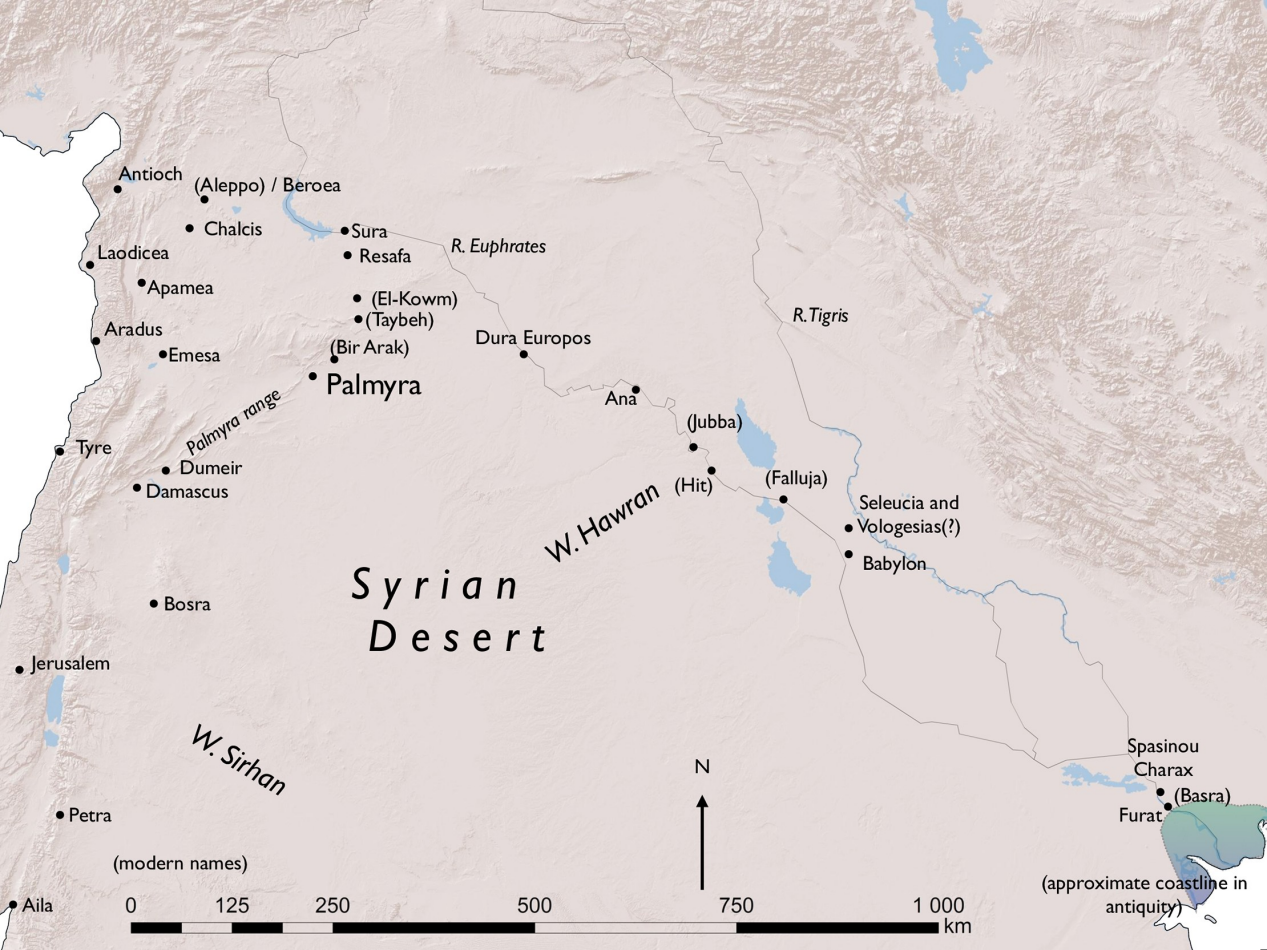
Map of Palmyra and the surrounding region. Reprinted with permission by professor Eivind H. Seland under a CC BY license. Basemap: ESRI.

The study of past societies is inherently based on incomplete data, often leading to criticism of such studies being subjective and widely open to interpretation. Within the social sciences, in particular archaeologists and ancient historians study societies to which they have no direct access through living individuals. Consequently, one of the pivotal challenges in these disciplines is to situate fragmented data as precisely and contextualized as possible within a wider cultural historical framework. While we will never be able to reconstruct how past societies worked in all details, we must strive to achieve as high an accuracy as possible through the evidence which we have at hand. We argue that it is crucial for archaeologists and historians to undertake methodological baseline studies based on large evidence samples from which broader theoretical studies can be undertaken. In order to exemplify such an approach and its significance, this paper addresses the largest body of material evidence depicting deceased people stemming from the ancient world, namely the Palmyrene funerary limestone portraits. These portraits were produced in Palmyra in the Syrian Desert over approximately 300 years, from about the beginning of the Common Era until the sack of the city in 273 CE (Fig 1). The fact that these portraits, were produced consistently across approximately 300 years, presents us with the possibility of studying the fluctuations in their production and evaluate whether they may be byproducts of economic and societal developments. Until now, they were predominantly regarded as a topic for art-historical research and have never been studied as a possible proxy for assessing the impact of historical events or processes, nor as a way of studying societal transformational patterns [1–4]. While it is not possible to demonstrate a direct causality link based on one dataset, this study establishes the first “benchmark” data trend, which can serve in the future as comparison for other, similar data analysis projects performed on other types of material culture evidence. Approaches in archaeology studying large and complex archaeological datasets when pulled together in one corpus and examined, on the one hand, and in archaeological and art-historical detail, on the other hand, within a larger framework of formal analysis methods are gaining popularity in Classical Archaeology [5–7], but they remain few and far between, not least because such detailed full-quantification studies–pulling together the entire known material of certain empirical group–demand extensive resources, which are often not available in projects within the humanities and social sciences, which traditionally work with legacy data, that is, the results and archival material of excavations before the 1960s, when the systematic collection of all material from an excavation began to be practiced widely, first in prehistoric archaeology [see 8, 9 for criticisms of this methodology and theory].

Here we demonstrate the potential of analyzing such large datasets of archaeological and art-historical significance to further a deeper and more nuanced understanding of past societal complexities–not only regarding the site from which the material comes, but also in a broader perspective. Such datasets enable comparison with historically attested events or even with archaeological material from other locations. As such, they can be used to better understand the long-term trajectories of human groups and the impact of different types of external factors, both negative perturbations and opportunities afforded by changing circumstances [e.g. 5, 6].

The objects of study have been collected since 2012 within the Palmyra Portrait Project [10–14]. They include limestone funerary portraits and portrait sculpture, the monumental tombs in which the portraits were set up and the human remains published from excavations in Palmyra [15]. Through the absolute and relative dating of the portraits we obtained an overview of the stylistic evolution of these portraits and the number produced over time, despite the fact that numerous have been lost [16, 17]. These datings enabled us to reconstruct flows and fluctuations in the portraits’ production trends and relate them to known and unknown historical events and processes, which brings entirely new knowledge about Palmyrene society’s resilience across centuries. The results presented here are the outcomes of interpreting the temporal fluctuations in funerary portrait production patterns in relation to historical events and underline the unleashed potential of studying large and complex archaeological datasets when pulled together in one corpus and examined, on the one hand, in archaeological and art-historical detail and, on the other hand, within a larger framework of formal analysis methods.

### Ancient Palmyra

Roman Palmyra, ancient Tadmor, was an oasis city in the Syrian Desert, which flourished in the first three centuries CE [18]. Palmyra was rediscovered by European travelers in the 17th century CE and gained much attention in European cultural circles in the 18th and 19th centuries [19, 20]. In particular in the late 19th century, the art of the city attracted attention, and this is the period in which the earliest Palmyrene collections in Europe came into existence [11]. Palmyra, since 1980 a UNESCO World Heritage site, is known today because of its good state of preservation, its extensive ruins stemming primarily from the Roman period and the devastation, which since 2012 has taken place in the wake of the escalating conflict in Syria [16, 17].

**Fig 2:**
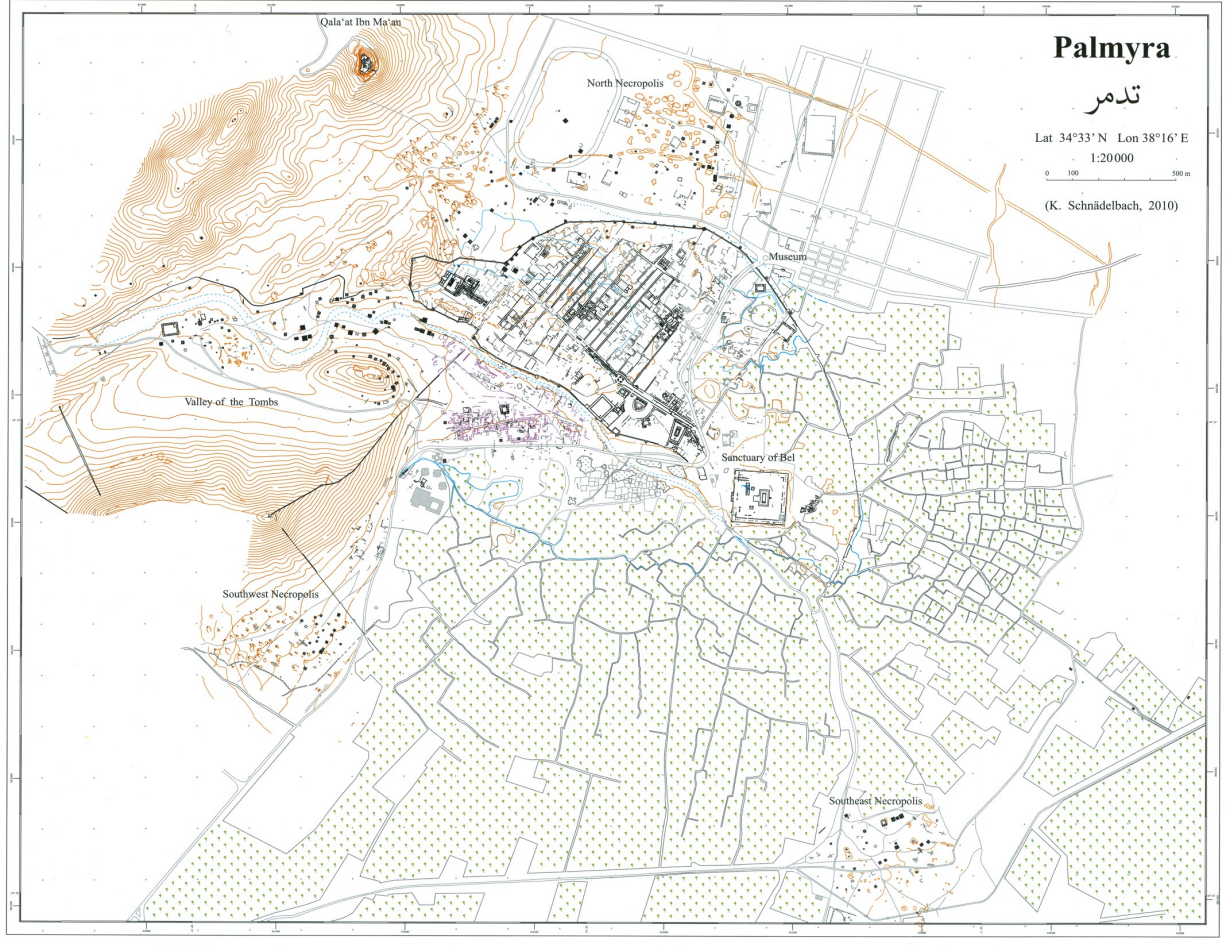
Map of Palmyra and immediate environs. Reprinted with permission by Klaus Schnädelbach, after Topographia Palmyrena, Damascus 2010.

The city was located halfway between the river Euphrates to the East and the Mediterranean Sea to the West [21, 22] (Fig 2). The nature of the site’s location with perennial access to water coming from the Efqa spring made the site ideal for settlement, and archaeological evidence shows that human activity had taken place there as early as the Paleolithic period [23–25]. However, as a monumentalized urban site, Palmyra only became truly graspable around the beginning of the Common Era [26]. With the Pax Romana (the Roman Peace), instituted after the Battle of Actium in 31 BCE, the Near East in general saw a renewed prosperity. Political and military stability led to economic upswings of unseen dimensions, and urban expansion across the region is documented in the centuries thereafter [26–28]. The economic upswing also made its impact on Palmyra, and at its largest extent in the second century CE the city covered about 120 hectares [29]. Palmyra was not, however, a metropolis, of which only few existed in Antiquity, such as Rome, Alexandria, and Antioch. Size-wise the city was located in the upper range of Roman-period middle-sized cities.

Palmyra was plainly put not more than an ordinary city, in an out-of-the-ordinary location, under influence of the Roman and Parthian cultural spheres, but with a strong Hellenistic and local heritage as the city’s bilingual inscriptions from the public sphere show [26, 29–33].

Palmyra benefitted particularly from the political stability in the region due to its location as a trading hub in the Syrian Desert connecting routes from East to West and acting as a stop-over for the caravan trade that the Palmyrene elite was heavily involved in and the city expanded in the first centuries CE [34–36]. Palmyrene society thrived on the caravan trade and the city also mobilized a strong army, which defended the city that was situated between Rome and Parthia and therefore geopolitically at times was in a fragile situation. Its army at times also participated in Roman military activities and units also travelled with the caravans to protect them on the dangerous stretches through the steppe desert [37]. As Palmyra’s wealth and military strength grew, the city expanded its territory immensely and in the late third century CE Palmyra tried to make itself independent from the Roman Empire [38, 39]. This led to the sacks of the city in 272 CE and 273 CE by the emperor Aurelian, and the city never rose again. After the devastation of the city, life did continue there, but on a much smaller scale, and a Roman legion was installed [40, 41].

Palmyra’s location–between the river Euphrates and the Mediterranean–at a nodal point and on the border of two strong and rivalling empires–the Roman and the Parthian–not only influenced the city’s wealth and military priorities, but also its culture, which is reflected in the art and architecture, the languages used as well as the religious cults of the city [13, 42]. The strong portrait habit is to be understood within this cross-cultural framework [43].

### The Palmyrene funerary portraits and funerary structures

Palmyra has for centuries been known for its funerary portrait tradition as well as its distinct grave towers, and its rock-cut underground graves as well as its smaller, lavish temple/house tombs, dotting the landscape around the city [19, 20, 43–49]. The portraiture constitutes the largest group of funerary representations stemming from one location in the ancient world, factors which make the corpus the most significant portrait material from the entire ancient classical world. The funerary portraits depict men, women, and children of the city’s elite, a small but absolutely significant segment of the city’s society. Because of Palmyra’s dependence on long-distance trade and the difficult geopolitical location between the two highly militarized empires as well as a strong tribe-based organization of the society, the elites held the key to the city’s wealth and its political, religious, and social structure [30, 50–53]. Thus the elite, and therefore the representations of them in the funerary sphere across three hundred years, can be regarded as a good proxy for approaching the economic, social, cultural, and political history of Palmyra–however, only if studied as a collective group as done here. The portraits were usually set up in monumental funerary structures, tower tombs, underground graves, and house or temple tombs (see below). A few examples were also placed on the outside of funerary structures.

### The Palmyrene funerary portraits

Palmyrene funerary reliefs, commonly referred to as Palmyrene portraits, are depictions of deceased carved on local limestone slabs, which were located in front of the burial shelves, the so-called loculi, in the graves functioning as grave markers. They were predominantly busts, more rarely entire figures, and could depict one or multiple individuals. In the later second century CE limestone sarcophagi were introduced on the lids of which family dining scenes (banquets) were depicted and on the boxes further portraits could be carved.

The portrait habit in Palmyra was introduced around the beginning of the Common Era and drew on a renewal of earlier Hellenistic traditions in the region, which were revived with the stronger presence of the Romans [13, 19, 54]. The production cannot be traced beyond the last sack of the city in 273 CE [1, 18]. The portraits therefore provide us with a *terminus post quem* and a *terminus ante quem*. This is a unique situation for archaeologists and art-historians, and combined with the amount of portraits we have to do with an outstanding corpus of material on the basis of which solid statistical analysis can be undertaken.

**Fig 3:**
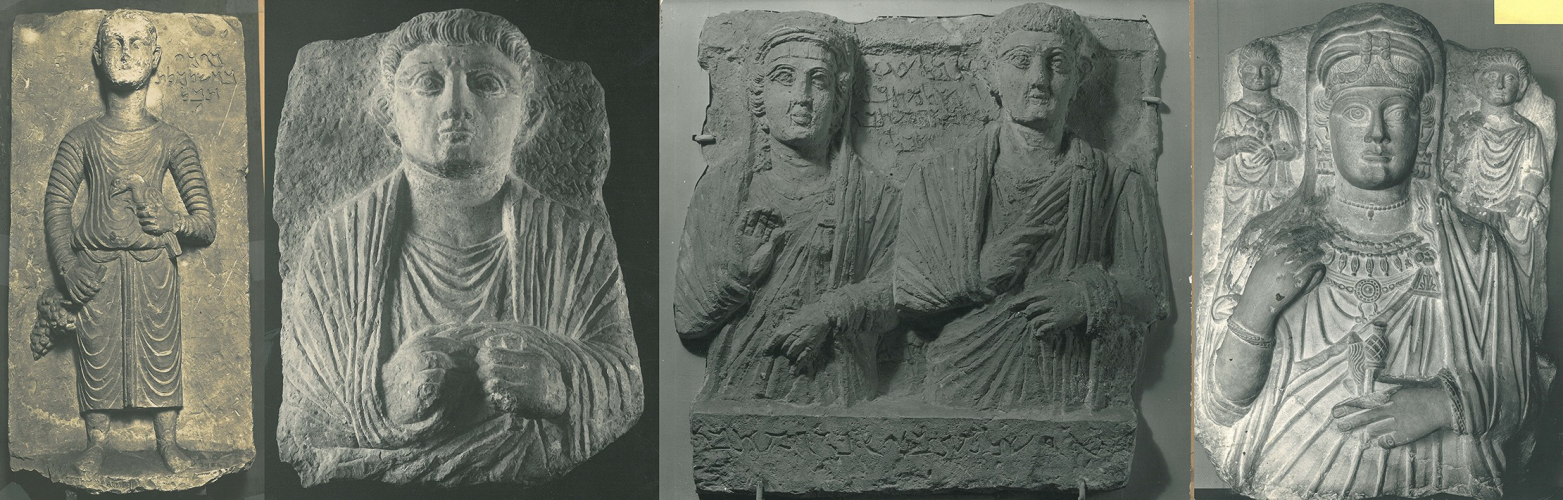
Loculus reliefs: Stele depicting a boy, Musée du Louvre, inv. no. AO 3984; relief depicting a male bust, National Museum of Damascus, inv. no. Dam 19; relief depicting two siblings, Antikensammlung der Friedrich-Alexander Universität Erlangen, inv. no. 1184; relief depicting a mother and her two children, Arthur M. Sackler Museum, Harvard University, inv. no. 1908.3. All images © Palmyra Portrait Project, Ingholt Archive at Ny Carlsberg Glyptotek.

The Palmyrene funerary portraits come in a variety of types [19, 55]. The most common type is the so-called loculus relief, a rectangular limestone slab upon which one portrait or more were carved in high relief (Fig 3). Another type is the so-called stele motif, also a rectangular limestone slab on which full-body or under-life-size representations were carved in high relief. Banqueting reliefs, showing dining scenes, constitute a third type [19, 52, 53, 56, 57]. All these types of reliefs were used to cover burial niches in the Palmyrene tower tombs, underground graves as well as in the temple or house tombs. The earliest funerary portraits appear on stelai [58], however the popularity of this type decreases with the appearance of the loculus reliefs, the earliest of which is dated by inscription to 65/66 CE [18, pp. 66–67, cat. 1]. Most of the surviving examples of stelai and banqueting reliefs are in museum collections outside of Syria, with no information about their in-situ provenance, but the few objects that were excavated in the twentieth century, show that both types were not very common. The Palmyra Portrait Project has catalogued 157 stelai and 99 banqueting reliefs. They span all three centuries of Palmyrene portraiture, with most stelai dated in the first and second centuries CE, whereas most banqueting reliefs are dated in the second and third centuries CE. Sometimes elaborate versions of banqueting reliefs were also set up on the facade of tower tombs as founder reliefs [46] or in prominent niches in the graves. These reliefs underlined the importance of the owner of the tomb, but were not associated directly with the burial [59].

**Fig 4:**
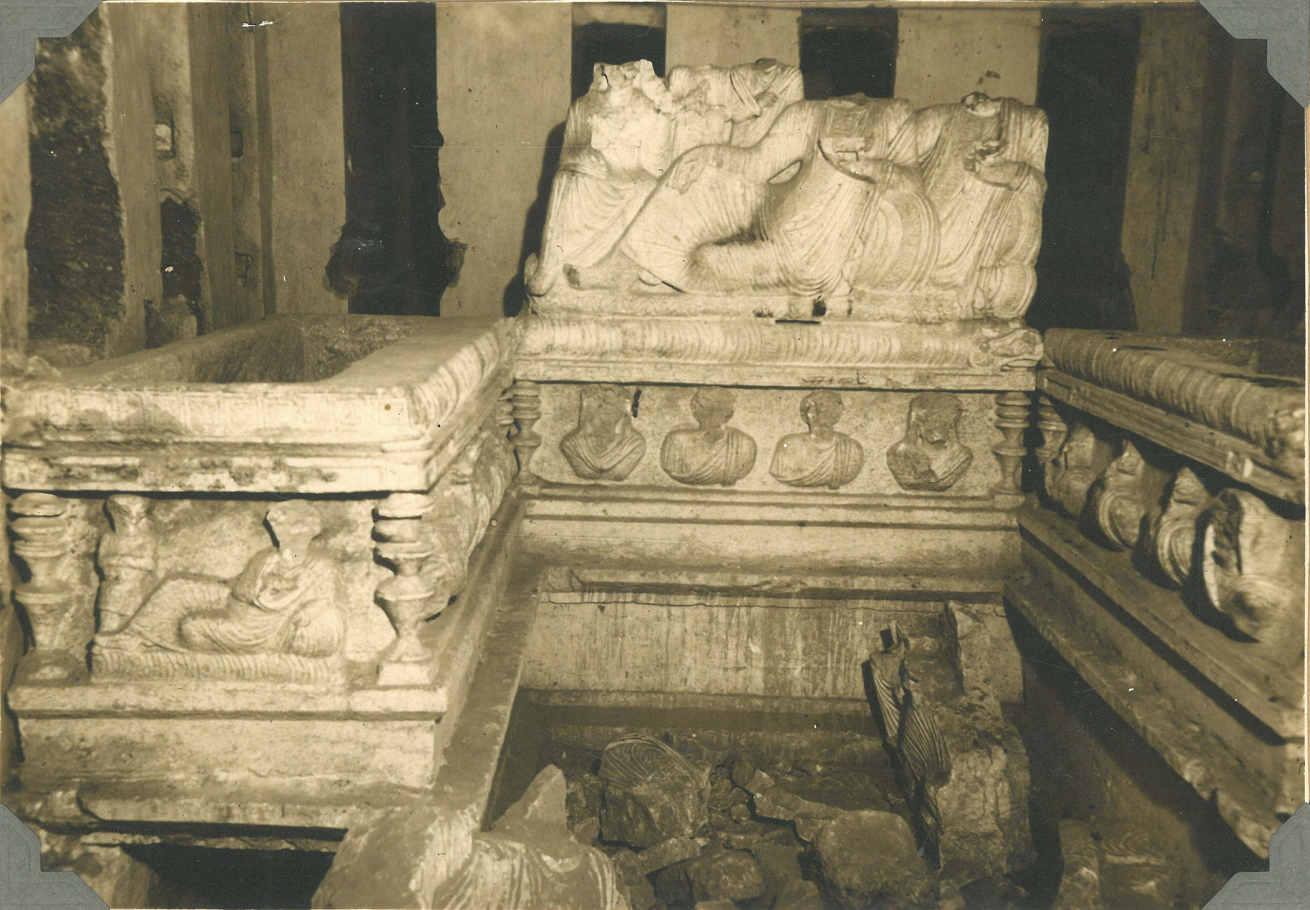
Sarcophagi from the hypogeum of the three brothers. Image © Palmyra Portrait Project, Ingholt Archive at Ny Carlsberg Glyptotek.

In the later second century CE, sarcophagi (burial boxes with relief lids), also made of the local limestone, were introduced in Palmyra. These were set up in the underground graves and the temple or house tombs. The sarcophagi could carry lavish banqueting scenes with many individuals shown together on their lids, and several portraits were depicted on the boxes [52, 60] (Fig 4). The sarcophagi commemorated entire families by depicting scenes of families at banquets [19, 43, 52]. Sarcophagi could hold numerous burials.

The portraits from Palmyra show a strong continuity in the local cultural and societal identity. While drawing on Roman models as well as being influenced by Eastern traditions from the realm of the Parthian Empire [61–64], the Palmyrene portraits were entirely adapted to the local burial custom, namely to be used as commemorative monuments in the monumental grave buildings. The portrait style and the focus on the more-than-bust-sized representations of the individual as well as the full-figures on the later sarcophagi lids stayed consistent across all three centuries of production.

However, distinct stylistic developments and fashion trends, which can be pinned down chronologically due to comparative material from Roman and Parthian material, can be detected in the portraiture, including in for example the dress, jewelry, and hairstyle fashions as well as the attributes carried by the individuals, underlining that Palmyrene society–at least within the elite–adhered to a collective and deep-rooted societal value system, which was influenced also by the changing world outside Palmyra. These factors reflect influences from the East and the West [63, 64], but in general the styles adhered to local customs, which in turn underlines the strong and continuous focus on Palmyrene society’s own identity based on a tribal environment as known from written sources. The changing fashions reflected in these attributes and other stylistic transformations of the portraits enable us to determine their age with a high degree of accuracy.

### The Palmyrene funerary structures

**Fig 5:**
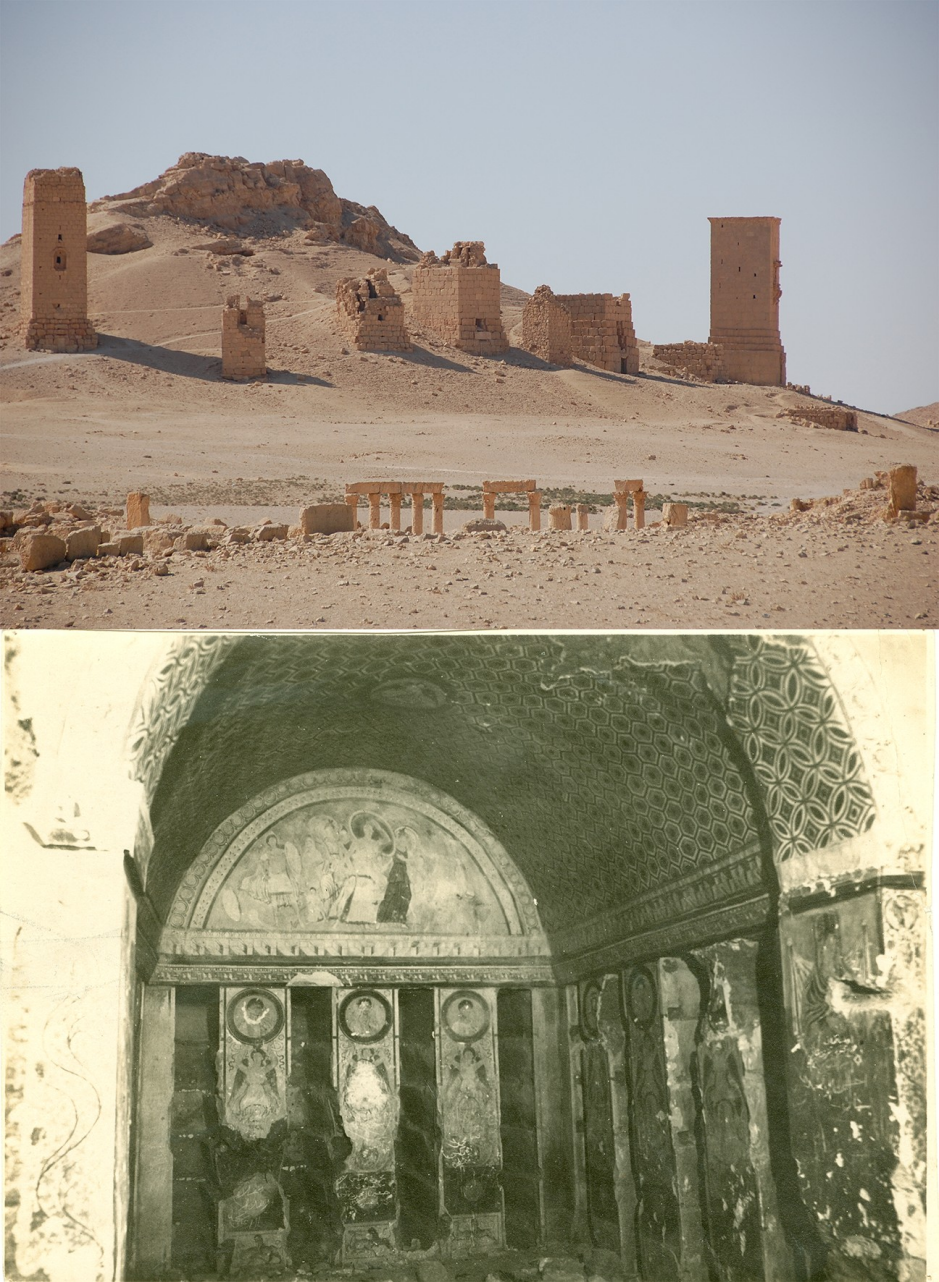
Upper row: View of tower tombs. Image © Rubina Raja. Lower row: view of the Hypogeum of the Three Brothers. Image © Rubina Raja and Palmyra Portrait Project, Ingholt Archive at Ny Carlsberg Glyptotek.

Several hundred tomb buildings have been discovered in the city [65]. In Palmyra three types of funerary monuments for the elite existed. The so-called tower tombs [46, 47], the underground tombs (hypogea) [70, 72] as well as the somewhat smaller but still monumental house or temple tombs [44] (Fig 5).

**Fig 6:**
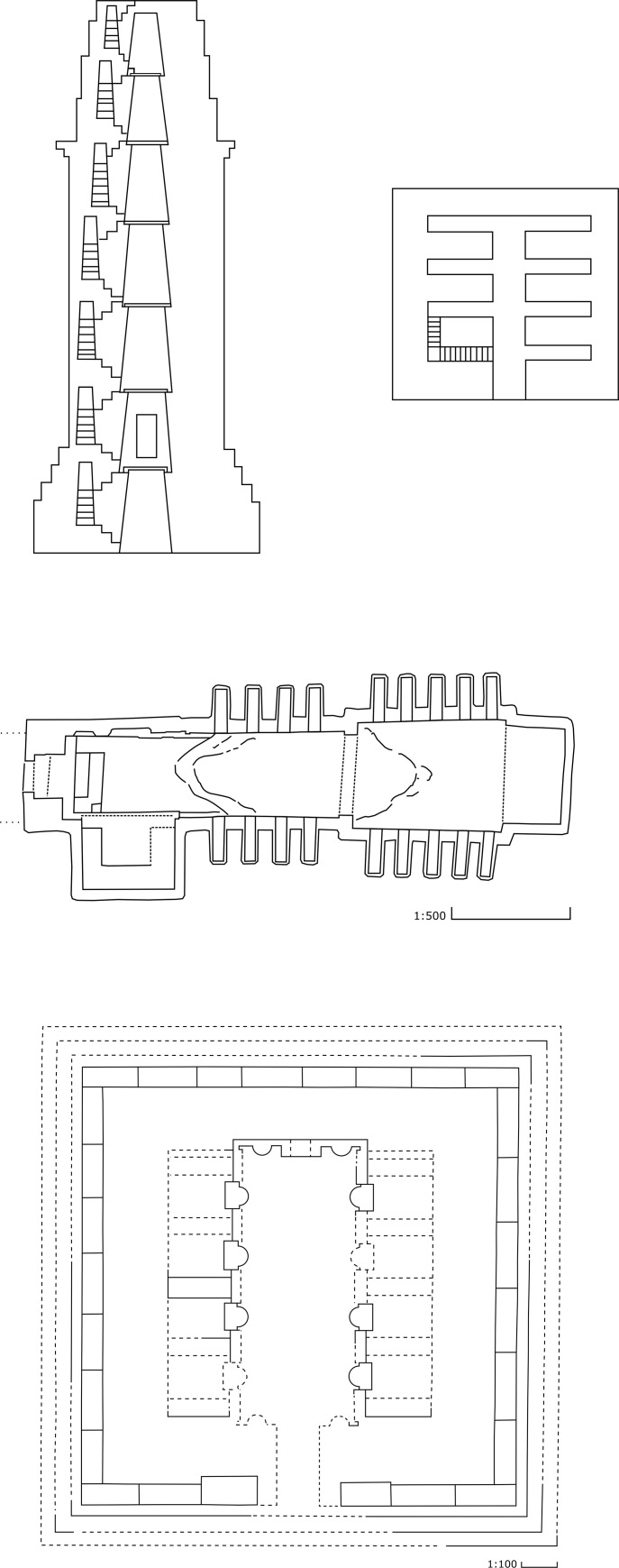
Plans of funerary monuments: Upper row: Tower tomb no. 51. Middle row: Hypogeum of ‘Atenatan. Lower row: ‘Qasr-Abjad’ temple tomb. All drawings © Palmyra Portrait Project.

The tower tombs were introduced in the early first century CE followed by the underground tombs and the house or temple tombs (Fig 6). The tower tombs were tall buildings in several stories, which could house hundreds of burials [44, 46, 47]. These tombs are distinct to Palmyra and very few tower tombs are found outside Palmyra [46, 65, 66; for tower tombs, see 67, 68: esp. pp. 52–106]. Research has shown that the first monumental tombs in Palmyra were the tower tombs, located mostly on the hills of the city’s west necropolis. The earliest of these is dated to 9 BCE, while the latest tower tomb was founded in 128 CE [46: p. 3]. The evidence of a grafitto found inside one of the tower tombs, however, indicated that they were in use until the third century CE [46: p. 92]. In the early first century CE, the founders began building underground tombs associated their tower tombs [44: pp. 60–68].

The underground tombs, also known as hypogea, were cut in the rock and accessible through a single staircase (a dromos) leading down to the tomb, which would have a central aisle and most often a set of side aisles and niches. The underground tombs had the advantage that they could be expanded over time as seen fit, since side chambers and further side aisles could be cut if the main aisle was filled up by burials [for underground tombs see 44: esp. pp. 107–28, 69–72]. The first underground tomb built independently of a tower tomb is dated to 81/82 CE [44: p. 48, 49: pp. 155–72]. These graves were dug into the bedrock, and were usually in the shape of an inverted T. The tomb of the founder was usually placed in a chamber or recess facing the door, so that it would be immediately visible upon entrance [49: pp. 155–72, esp. pp. 164–66].

The third monumental tomb type is that of the temple or house tomb, thus called either because of its temple-like façade with pillars or columns articulating its structure on a podium with or without a pediment, or because it contained an inner peristyle courtyard, like contemporary houses [49: pp. 155–72, esp. pp.166-70, 73]. The house or temple tombs could also be in several stories, but were much smaller than the tower tombs and most likely restricted to the upper layers of the Palmyrene elite [53] [for house or temple tombs see 44: esp. pp. 129–46, 45, 74, 75]. The earliest of these was dated to 143 CE [49: pp. 155–72, esp. p. 170]. Thus, hypogea and temple or house tombs replaced tower tombs as the preferred types of monumental tomb from the middle of the second century CE, although tower tombs continued to be in use until the third century CE.

The monumental graves, reserved for the elite, were usually founded by and paid for by a family father. They would be maintained by following generations, and sometimes the foundations are dated by inscriptions giving further fix points to our chronology. Legal texts inscribed on some of the graves inform us that parts of graves could be sold off to other families [76], and there is also written evidence underlining that older burials could to be taken out of the graves and new ones installed [77], making the tombs dynamic spaces. Other groups of inhabitants would have been buried in more insignificant graves, such as single-shaft graves without any sort of lavish embellishments [78].

The monumental funerary monuments give insight into Palmyrene burial traditions and rituals. The Palmyrenes practiced inhumation throughout the period under investigation by the Palmyra Portrait Project [65: p. 111, 79, 80]. Bodies were wrapped in textiles, perfumed and oiled, and placed on burial shelves in the niches and in the sarcophagi [79, 80]. Cremation was exceedingly rare, with only two examples known [69: esp. p. 256, 81: esp. p. 151]. Another rare practice testified in Palmyra, and only attested in the tower tombs, is that of mummification [65: p. 111 with previous bibliography]. Wells, lamps, and cooking vessels among other finds testify to rituals associated with the burial, or commemoration of the deceased [65: p. 152].

## Materials and methods

### Data collection

Today the Palmyrene funerary portraits are scattered in museums and collections in Syria and other parts of the world, since many were exported from the late 19th century onwards, when western interest in the cultures of the Near East surged [11]. Therefore, the research which has gone into collecting these portraits was time-consuming. The largest collection of the portraits outside Syria is held by the Ny Carlsberg Glyptotek in Copenhagen [18], closely followed by the National Museum in Istanbul [82–84] and the collection at the Louvre [85]. However, numerous museums and collections hold one or a few objects, which often have not been published or have been published in print publications only (for example, two reliefs whose present location is unknown, but were documented in Mary, Turkmenistan [86]; and single Palmyrene loculus reliefs in Australia and the USA [87–90]).

Since 2012 the Palmyra Portrait Project (https://projects.au.dk/palmyraportrait/) has compiled a database now holding 3704 portraits, and 329 tomb buildings and 769 individual burials based on material in collections and publications [13]. The majority of the portraits descriptions were compiled using available published material, however, approximately 2000 were investigated in person at different levels of details according to their location–on display or in storage–at numerous collections across the world, including Ny Carlsberg Glyptotek and National Museum of Denmark (Denmark); Musée du Louvre, Musée Rodin, Institut du Monde Arabe (all Paris, France); Staatliche Museen zu Berlin, Bode Museum Berlin, Glyptothek Munich, Ikonenmuseum Recklinghausen, Antikensammlung im Martin von Wagner Museum Würzburg (Germany); Bible Land Museum, Musée de Sainte-Anne, The Israel Museum, Rockefeller Museum, Terra Sancta Museum, W.F. Albright Institute of Archaeological Research (Israel); Musei Vaticani, Museo di Scultura Antica Giovanni Barracco, Fondazione Dino ed Ernesta Santarelli (Italy); American University Museum Beyrouth, Musée National de Beyrouth, Robert Mouawad Private Museum, Syriac Catholic Patriarchate Beyrouth (Lebanon); National Museum of Damascus, Palmyra Museum (Syria); Rijksmuseum van Oudheden (The Netherlands); Antakya, Gaziantep Arkeoloji Müzesi, Gorgo Medusa Cam Eserleri Müzesi, Hatay Arkeoloji Müzesi, Istanbul Arkeoloji Müzesi (Turkey); Ashmolean Museum, British Museum (England). At present, 3704 Palmyrene funerary portraits divided on 2713 objects have been compiled in a database designed specifically for the use of the Palmyra Portrait Project [131, 142]. Each type of object is entered into the database and thereafter split up into numbers of portraits. Images are included in the database for by far most of the objects. 33 objects in the database are only known from the literature and were originally published without images. The dating of these cannot be verified by the research team, since they have not been seen. The objects and portraits are described in detail, recording measures and all details of the objects and portraits, including facial and body features as well as attributes, such as clothing, jewelry, and items carried by the deceased [91]. Furthermore any given context information is added. The stylistic and chronological developments of the portraits have been studied on the basis of art-historical criteria [1, 2, 92] and the inscriptions which often accompany the portraits, whereof 4.2% give exact years of death, as well as in-situ portraits [93, 94].

### Chronology: Absolute dating

Most of the portraits have over time been removed from their original contexts through reuse, looting, vandalism, and destruction, which has taken place since Late Antiquity. Despite the fact that most are today ex situ, the almost 4,000 portraits make up a statistically solid group, datable, on the one hand, by fixed points given by objects dated by inscriptions [141, Table 1] and, on the other hand, by stylistic dating [141, Tables 2–4]. The absolute dating of the objects is based on 101 objects with reference to individuals that are dated to a specific year through their accompanying inscriptions [141, Table 1]. Other portraits and portrait sculptures are dated on the basis of careful observation of stylistic developments, which is a method widely applied within classical archaeology and art-history and which for the Palmyrene material has been further developed within the Palmyra Portrait Project [141, Tables 2–4; also see 1, 93]. Furthermore, these observations are held up against the absolutely dated portraits with inscriptions and the in-situ contexts. These parameters are those well-established and widely used within classical archaeology and art historical approaches [i.e. 1, 62, 92, 95, 96]. Most of the stelai that can be associated with a known funerary monument come from underground tombs, for example, 1) stele with standing male and female [71: p. 81, cat. 110, Fig 11]; 2) stele with standing boy [71: pp. 73–74, cat. no. 95, Fig 15]; 3) stele with standing male and child [71: pp. 85–86, cat. no. 119, Fig 19]; 4) stele with standing child [97: pp. 78–79, 132, no. 6, pl. 49–50]; 5) stele with standing boy [97: pp. 130–32, no. 5, 77–78, pl. 48, 9, Fig 15]; 6) stele with standing child [79: p. 104, Fig 72-S2, pl. 63,3]; see also [53]. Only one stele can be securely connected to a tower tomb, tower tomb no. 68, of Bene Baa [46: p. 211, pl. 60b]. Banqueting reliefs were rarely placed in tower tombs, but see 1) tower tomb no. 13, of Elahbel, banquet relief with two figures [46: pp. 306–07, cat. S 46, pl. 27, c]; 2) tower tomb no. 67, of Ḥairan [46: p. 208, pl. 59a]), and more often in underground tombs, for example, 1) Hypogeum of Taîbbôl, banqueting relief with four figures [98: p. 132, Fig 10]; 2) Hypogeum of the Bôlbarak family, banqueting relief with two figures: Palmyra Museum, Palmyra, inv. no. 1793/6642 [71: pp. 145–46, cat. 193, Fig 253]; 3) Hypogeum of Zabdâ, banqueting relief with two figures: Palmyra Museum, Palmyra, inv. nos. B 2024/7222 and B 2025/7223 [71: pp. 138–39, cat. no. 184, Fig 220–21]). Only one example is documented from a temple or house tomb, that of A’ailamî and Zebidâ, (“Tomb Cantineau”) [99: p. 87, Fig 9]. For banqueting reliefs, see [52, 74, 100, 101].

### Chronology: Stylistic dating

The 156 portraits dated by inscriptions form a cornerstone of our research, since they indicate what particular stylistic features, clothes, and attributes were popular in the years that they were created. Another 661 portraits are dated with a high relative certainty, since they come from tombs that were in use for specific decades. The rest of the portraits are dated based on comparison with these 817 portraits that can be dated either within a year or a decade thanks to their inscription or find location. Thus, although we cannot reconstruct the in-situ situations of the majority of the portraits, it has no impact on the stylistic study undertaken of the portraits, since this is based on stylistic developments and dated using the portraits with absolute dates attached to them [1–3].

The stylistic analysis of these portraits, undertaken by at least four researchers from the Palmyra Portrait Project per portrait and compared to previous analyses by classical archaeologists and art-historians [1, 2, 62, 92, 95, 96], allowed identification of a number of attributes and sets of features whose appearance is circumscribed to a certain chronological period. These attributes, most of which are related to the specific technique for depicting eyes, facial hair, textiles, jewelry or weapons, etc. allowed the researchers of the Palmyra Portrait Project to date the portraits and place them within specific groups (only a brief list of the attributes and techniques that allowed for chronological phasing is provided). A concise table with all the criteria used to ascribe each portrait to one phase or another is included in the supplementary materials [141] and in detail describe the stylistic features and their chronological fix points and spans as well as give reference to earlier published research.

This analysis and the identification of them allowed us to define seven phases with a specific chronological range. Each of these phases covers a chronological range of varying but not overlapping duration. The length of the phases is determined by the number of absolutely and relatively dated portraits with similar features: the first period (1–100 CE) is relatively long, as only 11 absolutely dated portraits are known from this period, all featuring some common elements regardless of the function of the objects. The next periods are shorter, as the number of firmly dated portraits increases after 100 CE, allowing for greater refinement of the dating criteria, and for changes in style to become more apparent. At the same time, after the end of the first century CE, the number of relatively dated portraits also increases, thus enlarging the group of portraits that can be used as the basis for stylistic dating. For the portraits in the last phases especially, the evidence of the legal texts from tombs is as useful as is that of the foundation inscriptions, as these make it possible to date groups of portraits within specific decades (for example, the portraits from the west exedra from the hypogeum of Yarḥai can be dated securely within the period 240–273 CE, since the text recording the sale of that particular area of the hypogeum in the year 240 survives: 69)

### Period I. 1–100 CE

This is the period of the earliest attested portraits in Palmyra. They appear in stelai and loculus reliefs. In stelai, they are shown as full figures, whereas in loculus reliefs, when only one person is depicted, then only the area above the waist is shown. If there are several people depicted in the loculus relief, then there is a differentiation between adult figures, shown from above the waist, and children, shown either to the side of the adult in full figure, but smaller in scale, or behind the shoulders of the adult. The iconography of children is consistent throughout the different periods under study: they are shown with short hair, and holding birds and usually bunches of grapes, less often dates, or writing tablets. They usually wear tunics, and less often, Parthian-inspired tunics and trousers [102: pp. 67–76]. In this period, the first founder and banqueting reliefs appear as well, introducing the motif of the reclining male in a banquet together with his family. The reclining male takes pride of place. In reliefs with a banqueting motif of this period, he is to be identified with the founder of the tomb. In the earliest of these reliefs, from the tower tomb of Kitôt, the female accompanying the male founder, identified as his wife thanks to the inscription, is shown standing [46: p. 161, 163–64, Figs 4, 15; 102: esp. 568, Fig 5]. This is a unique representation that is not repeated in the other banqueting reliefs, where the wife is shown seated.

Males are depicted with short hair that is not very defined (so, only with few thick locks or snail-shell curls of hair without detailed rendering of many strands) [103]. The eyebrows are shown as curving grooves, and the irises and the pupils are indicated by concentric, incised circles [104]. All the men are beardless [103]. They wear a plain tunic [103, 104] under a himation, which can be depicted either with semi-circular folds [103] or in the ‘arm-sling’ type [105, 106]. The ‘arm-sling’ type is better known from Greco-Roman costumes: the himation is wrapped around the person’s body, with one end crossing the chest and falling behind the left shoulder, and the right arm placed inside the sling created by the draping of the himation over the chest and shoulder. This manner of wearing the himation is the typical Greco-Roman way of depicting men involved in the affairs of the city-state (that is, having the right to vote and being active participants in the debates concerning the city, if not also holding a political office), and probably had a similar connotation in Palmyra [107]. They usually hold one attribute: this can be a book-roll, held either in the left or the right hand [103, 105, 106], a leaf held in the left hand [103, 105], a sword held in the left hand, or a whip, also held in the left hand [103]. The book-roll has been associated with learning and as a marker of an educated man [108], while the meaning of the leaf eludes us [109]. The sword has immediate military connotations, while the whip has been associated with the camel-drivers of Palmyra [see also 110].

A particular sub-group of men is that of priests. Priests are characterized by their cylindrical and flat-topped hat. In this period, their hat is plain and undecorated [14, 43, 55, 105, 111–114]. It has been proposed that their heads were shaved [55, 111–115], and they are beardless [111, 113, 114, 116]. The rendering of the facial features of priests follows that of the other men: eyebrows traced by curving grooves, and eyes with irises and pupils indicated by concentric, incised circles. They wear a tunic that is sometimes decorated or embroidered [14, 43, 55, 105, 111, 113, 114, 116, 117] under a chlamys [14, 43, 55, 105, 111, 113, 114, 116, 117] that is fastened over the right shoulder with a circular brooch [43]. This elaborate costume has been associated with the Parthians [118, with previous bibliography], and even though it is not used in depictions of priests when performing their duties [119], it is commonly used for priests in funerary contexts [14, 43, 55, 105, 111, 113, 114, 116, 117]. They often hold a libation pitcher and incense bowl, which are often decorated [14, 43, 55, 105, 111, 113, 114, 116, 117].

Women in the same period appear with their hair almost fully covered by veils and headbands. The folds of the veil are rigid and vertical [88], while the headband that covers the forehead fully or partly is either plain or simply decorated with vertical grooves [88, 120]. Only two shoulder locks [51, 88], or a few locks of hair brushed back over the temples are shown [51, 87]. Their eyebrows can be depicted by curving ridges or grooves [88]. They wear a long-sleeved tunic with a rounded neckline under a himation with semi-circular folds [88]. Their jewelry is limited in number, and even though it is possible to have multiple types of jewelry (for example, necklace and earring and brooch), more often they wear only earrings and brooches. When they wear necklaces, they are composed of round beads, and usually worn in one strand [88]. There are three types of earrings that may be worn: the earliest is a series of small hoops that are worn on the helix [88]. Two types appear near the end of the century: they are earrings in the shape of bunches of grapes [88] and earrings in the shape of horizontal bars with two or three round hanging pendants [88]. The brooches are trapezoidal, and in some cases, there are keys hanging down from them [88, 120–122]. In their left hand, the women are shown holding a spindle and a distaff, the two attributes most connected to women’s work in the domestic sphere in the Eastern Mediterranean [88, 105, 120]. Their posture is frontal, with their head sometimes turned slightly. Their arms are shown either resting against the chest [88] or with the palm of the right hand held up and forward [88, 105].

### Period II. 100–135 CE

Funerary portraits continue to be depicted on stelai, loculus reliefs and banqueting reliefs. The conventions regarding the size and placement of figures observed in the previous period continue in this period, as well as in the subsequent ones.

Males are represented with short hair, but better defined: the curls, usually comma- or crescent-shaped, are arranged in rows over the head [4, 103]. The eyebrows and eyes continue to be rendered in the same manner as in the previous period: with curving grooves for the eyebrows, and with the irises and pupils indicated by concentric, incised circles [104]. In addition to the depiction of beardless males, men also appear with beards from this period onwards [4, 106]. The choices of costume and attributes show continuity with the previous period. Men are represented wearing a plain tunic [103, 105] under a himation rendered with semi-circular folds [103], or in the ‘arm-sling’ type [105, 106], and holding either book-rolls in the left or the right hand [103, 105, 106], or a leaf in the left hand [103, 105], or a sword or a whip in the left hand [103].

Priests continue to be represented wearing their cylindrical and flat-topped hat, but in this period the lower part of the hat is encircled by a wreath with a central decoration [14, 43, 55, 105, 111, 113, 114, 116]. Their heads were most likely clean-shaved [55, 111, 113–116] and they are beardless [111, 113, 114, 116]. They wear a tunic that is occasionally embroidered or decorated [14, 43, 55, 105, 111, 113–116] and a chlamys fastened over the right shoulder with a circular brooch [43]. They hold a libation pitcher and an incense bowl that are often decorated [14, 43, 55, 105, 111, 113–116]. So, while there is continuity in the costume and most attributes, a greater degree of elaboration can be seen in the priestly hat.

Females are shown as heavily veiled as in the previous period. Their headbands, however, are now decorated with rectangular panels that carry floral or vegetal motifs, and are separated by vertical beaded bands [88, 120]. The hairstyle shows greater elaboration: there are curving locks on both sides of the headdresses at the temples, as well as two shoulder locks [88]. The eyebrows are rendered by curving ridges or grooves [88], and the eyes have irises and pupils indicated by concentric, incised circles [88, 104, 120]. In addition to the continuity seen in the choice of depiction of facial features, there is continuity in choice of dress, jewelry, attributes, and posture. The women are depicted wearing a tunic that is long-sleeved and with a rounded neckline [88], under a himation rendered with semi-circular folds [88]. The jewelry chosen to be depicted are the same as in the previous period: necklaces composed of round beads; earrings: either a series of small hoops along the helix, or in the shape of bunches of grapes, or horizontal bars with two or three round hanging pendants [88]; and trapezoidal brooches, often used for the suspension of keys [88, 120–122]. In their left hand, they hold a spindle and distaff [4, 88, 105, 120]. Their posture is frontal, with their head sometimes turned slightly. Their arms are shown either resting against the chest [88], or with the palm of the right hand held up and forward [88, 105].

### Period III. 135–160 CE

In addition to funerary portraits in stelai, loculus reliefs and banqueting reliefs, including founder reliefs, sarcophagi also appear in this period. They are composed of a rectangular box for the burial of the deceased, decorated with portrait busts, while the cover is either flat with a relief depicting a banqueting scene placed at the edge, or it is sculpted in the round, and depicts a family in a banqueting scene. As in the previous periods, pride of place is given to the reclining male, who rests on a pillow at the left end of the relief, while the most important female in the family is shown seated at the right end. These are to be identified usually with the father and mother of the family, while their children are shown standing in between the two [52]. These reclining men may appear in a tunic and himation, or in a decorated long tunic and trousers, a costume inspired by Parthia [see 59, 118]. Another characteristic of this period is the appearance for the first time of inscriptions in Greek or Latin in the funerary sphere, in addition to the inscriptions in Palmyrene Aramaic [123].

Males continue to be depicted in the same way as in the previous periods. They are shown with short hair, with comma- or crescent-shaped curls arranged in rows over the head [4, 103]. The eyebrows are rendered by curving grooves [104], and the eyes have the irises and pupils indicated by concentric, incised circles [104], and they may be shown with or without a beard [4, 103, 106]. They wear a plain tunic [103, 105] under a himation rendered with semi-circular folds [103], or in the ‘arm-sling’ type [105, 106]. They also hold the same attributes as in the previous periods: a book-roll held either in the left or the right hand [103, 105, 106], a leaf in the left hand [103, 105], or a sword or a whip in the left hand [103].

The representation of priests also shows continuity with that of the previous periods. The priestly hat is encircled by a wreath with a central decoration [14, 43, 55, 105, 111, 113, 114, 116], and their heads were probably shaved [55, 111, 113–116]. They wear a tunic, occasionally embroidered or decorated [14, 43, 55, 105, 111, 113–116] under a chlamys [11, 44, 56, 71, 106, 113, 114, 116, 117] fastened on the left shoulder with a circular brooch [43]. They are shown holding a libation pitcher and incense bowl that are often decorated [14, 43, 55, 105, 111, 113–116].

Females are shown wearing veils. In this period, in addition to depiction of the stiff, rigid folds seen in the previous two periods, naturalistic vertical folds are also depicted [4, 88]. They are also shown wearing a turban under the veil, usually rendered as a piece of cloth wrapped around the head, usually three times, creating the impression of three layers of fabric, and a headband that is similar to that of the previous period, with rectangular panels decorated with floral and vegetal motifs and separated by vertical beaded bands [88, 120]. The hairstyle is the same as in the previous period: there are curving locks on both sides of the headdresses pushed back at the temples, as well as two locks that fall down on the shoulders [88]. The eyebrows are rendered by curving ridges or grooves [87], while the eyes are rendered in two ways: either with the irises and pupils indicated by concentric, incised circles, as in the previous period [88, 104, 120], or with the irises indicated by incised circles and the pupils indicated by punch holes [88]. There is greater variation in the clothes as well: the tunic can be either long-sleeved with a rounded neckline, or short-sleeved, while the himation is depicted as having semi-circular to pointed folds [88]. There is also greater variation in jewelry: in addition to the simple beaded necklaces, beaded necklaces with a central pendant are shown, as well as more elaborate necklaces composed of plaited, or twisted, or loop-in-loop chains with a central pendant [88]. There are four types of earrings: 1) series of small hoops along the helix, 2) in the shape of bunches of grapes, 3) horizontal bars with two or three round hanging pendants [88], and 4) dumbbell-shaped earrings [88,120]. The brooches are trapezoidal, from which keys are suspended on occasion [88, 120, 121, 122]. Women are still represented holding a spindle and a distaff in the left hand [4, 88, 105, 120], but from this period onwards, they are also shown holding the edge of the veil with either the left or the right hand [88], or a child in the left hand [88]. Their posture remains frontal, with the head turned slightly on some occasions [4, 88]. The arms are depicted resting against the chest [87], or with the palm of the right hand raised and held forward [88, 103, 106], as in the previous period, but they can also be shown with the hand (usually the right, less often the left) raised to the height of the shoulder or neck [88, 103, 106], or with the hand (usually the right) resting against the cheek (usually the left) [88, 103, 106].

### Period IV. 160–175 CE

The types of monuments, constellations, and the use of Greek and Latin inscriptions in the funerary sphere show continuity with those of the previous period.

The depiction of males changes in this period. The hair is depicted as longer and thicker [103], and men are shown either with or without a beard [4, 103]. They wear a plain tunic [103, 105] under an ‘arm-sling’-type himation [105, 106], and they hold either a book-roll in the left or the right hand [105, 106], or a leaf in the left hand [105].

The representations of priests also show continuity with that of the previous periods. The priestly hat is encircled by a wreath with a central decoration [14, 43, 55, 105, 111, 113, 114, 116], and their head is probably shaved [55, 111, 113–116]. They wear a tunic, occasionally embroidered or decorated [14, 43, 55, 105, 111, 113–116] under a chlamys [14, 43, 55, 105, 111, 113–116] fastened on the left shoulder with a circular brooch [43]. They are shown holding a libation pitcher and incense bowl that are often decorated [14, 43, 55, 105, 111, 113–116].

Females are usually shown veiled. The veil falls in a naturalistic way over the head and shoulders [88]. Under the veil, they wear a turban with three layers of fabric, and a headband with rectangular panels decorated with vegetal or floral motifs separated by vertical beaded bands [88]. When they are veiled, they are shown with two curving locks of hair on both sides of the headdresses at the temples and one shoulder lock (rarely two) over the shoulder [88]. On some reliefs, women are shown unveiled or partially veiled, with their hair drawn up, in the ‘Faustina’ hairstyle [4, 51]. The eyes are rendered either with the irises, or the irises and pupils indicated by concentric, incised circles, as in the previous period [88, 104, 120], or with the irises indicated by incised circles and the pupils indicated by punch holes [88]. They wear a short-sleeved tunic with a v-shaped neckline under a himation with pointed folds [88]. They are shown wearing the same types of jewelry as in the previous period: simple beaded necklaces, beaded necklaces with a central pendant, as well as more elaborate necklaces composed of plaited, or twisted, or loop-in-loop chains with a central pendant [88]. The earrings in the shape of small hoops no longer appear, while earrings 1) in the shape of bunches of grapes, 2) horizontal bars with two or three round hanging pendants [88], and 3) dumbbell-shaped earrings [88, 120] continue to be depicted. The brooches have a much greater variety; they can be trapezoidal, circular, or polygonal, from which keys are suspended on occasion [88, 120–122]. Women are still represented holding a spindle and a distaff in the left hand [4, 88, 106, 120], or holding the edge of the veil with either the left or the right hand [88], or a child in the left hand [88]. The turn of the head is more pronounced in this period [88]. They are usually shown with the hand (usually the right, less often the left) raised to the height of the shoulder or neck [88, 103, 106], or with the hand (usually the right) resting against the cheek (usually the left) [88, 103, 106].

### Period V. 175–220 CE

The types of monuments, constellations, and the use of Greek and Latin inscriptions in the funerary sphere show continuity with those of the previous period.

The depiction of males in this period shows continuities as well as breaks with the previous period. The hair is depicted as longer and thicker [103], and for the first time, the eyes are left blank and undifferentiated [104]. The irises and pupils were likely painted on. Males are shown either with or without a beard [4, 103]. They wear a tunic that can either be plain or decorated with broad bands (*clavi*) [103, 105] under an ‘arm-sling’-type himation [103, 106]. In some cases, they also wear a fringed mantle [124]. They hold either a book-roll in the left or the right hand [105, 106], or a leaf in the left hand [105].

The representation of priests also shows continuity with that of the previous periods. The priestly hat is encircled by a wreath with a central decoration [14, 43, 55, 105, 111, 113–116], and their head is probably shaved [55, 111, 113–116]. They wear a tunic, occasionally embroidered or decorated [14, 43, 55, 105, 111, 113–116] under a chlamys [14, 43, 55, 105, 111, 113–116] fastened on the left shoulder with a circular brooch [43]. They are shown holding a libation pitcher and incense bowl that are often decorated [14, 43, 55, 105, 111, 113–116].

Females in this period are usually shown veiled. The veil falls over the head in naturalistic folds, often with a pleated, scalloped, zigzag, or woollen fringe [88], or it falls over the shoulders [88]. The turban and the headband are shown as in the previous period: a three-layered turban over a headband with rectangular panels decorated with floral and vegetal motifs separated by vertical beaded bands [88]. The locks that curve backwards over both sides of the headdress at the temples are thick and voluminous, while there is usually one, on occasion two, shoulder locks falling over the shoulder [88]. Women are also represented with the ‘Faustina’ hairstyle [4, 51]. The eyes are shown in different ways: 1) with the irises indicated by incised circles [88], 2) the irises and the pupils indicated by concentric, incised circles [88], 3) the irises indicated by concentric, incised circles and the pupils indicated by punch holes [8], or 4) they are left blank and undifferentiated [88, 123]. They wear a short-sleeved tunic with a v-shaped neckline under a himation with pointed folds [88], while on some occasions, they also wear a fringed mantle [124]. There is great variety in the jewels that may be depicted. It is possible to distinguish between five main types of necklaces: 1) beaded necklaces, often with a central pendant [88], 2) necklaces composed of plaited, or twisted, or loop-in-loop chains with a central pendant [88], 3) plain necklaces with one or two pendants [88], 4) necklaces with circular and diamond-shaped bezels linked by beaded elements [88], or 5) chain necklaces with medallions [87]. The earrings can be either 1) in the shape of bunches of grapes [88], 2) in the shape of horizontal bars with two or three round pendants [88], or 3) dumbbell-shaped [88, 120]. The brooches are trapezoidal, circular, or polygonal, occasionally with keys suspended by them [88, 121]. For the first time, women are also shown wearing bracelets. They can be either twisted and beaded [88], or with a bell [88]. They are represented holding a spindle and a distaff in the left hand [4, 88, 106, 120], or holding the edge of the veil with either the left or the right hand [88], or a child in the left hand [88]. They are usually depicted turning their head in this period [88]. They are usually shown with either the left or the right hand raised to the height of the shoulder or neck [88, 103, 106, 120], or with the left or right hand resting against the cheek [88, 103, 106, 120].

### Period VI. 220–240 CE

The types of monuments, constellations, and the use of Greek and Latin inscriptions in the funerary sphere show continuity with those of the previous period. There are two new additions in Palmyrene iconography: the motif of reclining women taking pride of place in banqueting reliefs (for example, a relief in Istanbul, Archaeological Museum, inv. no. 3728/180) [88: cat. 788], and scenes related to trade, caravans, or religious activities in the boxes of sarcophagi (see, for example, a sarcophagus from the Hypogeum of Atenatan, exedra of Julius Aurelius Maqqai) [77: esp. p. 67, pls. 26–27].

Males are usually shown with long, thick hair, but short hair is also worn on occasion [103]. The eyes are left blank and undifferentiated [104], and they are shown sporting beards [103]. They wear a tunic that can either be plain or decorated with broad bands (*clavi*) [103, 105] under an ‘arm-sling’-type himation [105, 106]. In some cases, they also wear a fringed mantle [124]. They continue being depicted with either a book-roll in the left or the right hand [105, 106], or a leaf in the left hand [105], but in some cases, they are shown holding a fold of the himation in their left hand [103].

The representation of priests also shows continuity with that of the previous periods. The priestly hat is encircled by a wreath with a central decoration [14, 43, 55, 105, 111, 113, 114, 116], and their head is probably shaved [55, 111, 113–116]. They wear a tunic, occasionally embroidered or decorated [14, 43, 55, 105, 111, 113–116] under a chlamys [14, 43, 55, 106, 111, 113–116] fastened on the left shoulder with a circular brooch [43]. They are shown holding a libation pitcher and incense bowl that are often decorated [14, 43, 55, 105, 111, 113, 114, 118].

Females in this period are usually shown veiled. The veil falls over the head in naturalistic folds, often with a pleated, scalloped, zigzag, or woollen fringe [88], or it falls over the shoulders [88]. The turban and the headband are shown as in the previous period: a three-layered turban over a headband with rectangular panels decorated with floral and vegetal motifs separated by vertical beaded bands [88]. The locks that curve backwards over both sides of the headdress at the temples are thick and voluminous, while there are occasionally two shoulder locks falling over the shoulder [88]. Women are also represented with the ‘Faustina’ hairstyle [4, 51]. The eyes are shown either with the irises indicated by incised circles [67], or they are left blank and undifferentiated [88, 123]. They wear a short-sleeved tunic with a v-shaped neckline that is often decorated, or a fringed, long-sleeved tunic [124] under a himation with pointed folds [88], while on some occasions, they also wear a fringed mantle [124]. There is great variety in the jewels that may be depicted. It is possible to distinguish between five main types of necklaces: 1) beaded necklaces, often with a central pendant [88], 2) necklaces composed of plaited, or twisted, or loop-in-loop chains with a central pendant [88], 3) plain necklaces with one or two pendants [88], 4) necklaces with circular and diamond-shaped bezels linked by beaded elements [67], and 5) chain necklaces with a medallion [88]. The earrings are dumbbell-shaped [88, 120]. The brooches are circular or polygonal [88, 121]. The bracelets can be either twisted and beaded [88], or with a bell [88]. They are represented holding the edge of the veil with either the left or the right hand [88]. The turn of the head is frequent in this period [88]. They are usually shown with either the left or the right hand raised to the height of the shoulder or neck [88, 103, 106, 120], or with the left or right hand resting against the cheek [88, 103, 106, 120].

### Period VII. 240–272 CE

Males are shown usually with long, thick hair, but short hair is also worn on occasion [103]. The eyes are left blank and undifferentiated [123], and they are shown sporting beards [103]. They wear a tunic that can either be plain or decorated with broad vertically running bands (*clavi*) [103, 105] under an ‘arm-sling’-type himation [105, 106]. They continue being depicted with either a book-roll in the left or the right hand [105, 106], or a leaf in the left hand [105], but in some cases, they are shown holding a fold of the himation in their left hand [103].

The representation of priests also shows continuity with that of the previous periods. The priestly hat is encircled by a wreath with a central decoration [14, 43, 55, 105, 111, 113–116], and their head is probably shaved [55, 111, 113–116]. They wear a tunic, occasionally embroidered or decorated [14, 43, 55, 105, 111, 113–116] under a chlamys [14, 43, 55, 104, 111, 113–116] fastened on the left shoulder with a circular brooch [43]. They are shown holding a libation pitcher and incense bowl that are often decorated [14, 43, 55, 106, 111, 113–116].

There are continuities in the depictions of women of this and the previous period. The veil falls over the head in naturalistic folds, often with a pleated, scalloped, zigzag, or woollen fringe [88], or it falls over the shoulders [88]. The turban is three-layered and worn over a headband with rectangular panels decorated with floral and vegetal motifs separated by vertical beaded bands [88]. The locks that curve backwards over both sides of the headdress at the temples are thick and voluminous, while there are occasionally two shoulder locks falling over the shoulder [87]. Women are also represented with the ‘Faustina’ hairstyle [4, 51]. The eyes are shown either with the irises indicated by incised circles [88], or they are left blank and undifferentiated [88, 123]. They wear a short-sleeved tunic with a v-shaped neckline that is often decorated, or a fringed, long-sleeved tunic [124] under a himation with pointed folds [87], while on some occasions, they also wear a fringed mantle [124]. There is great variety in the jewels that may be depicted. It is possible to distinguish between five main types of necklaces: 1) beaded necklaces, often with a central pendant [88], 2) necklaces composed of plaited, or twisted, or loop-in-loop chains with a central pendant [88], 3) plain necklaces with one or two pendants [88], 4) necklaces with circular and diamond-shaped bezels linked by beaded elements [88], and 5) chain necklaces with a medallion [87]. The earrings are dumbbell-shaped [87, 118]. The brooches are circular or polygonal [88, 119]. The bracelets can be either twisted and beaded [88], or with a bell [88]. They are represented holding the edge of the veil with either the left or the right hand [88]. The turn of the head is frequent in this period [88]. They are usually shown with either the left or the right hand raised to the height of the shoulder or neck [88, 103, 106, 120], or with the left or right hand resting against the cheek [88, 103, 106, 120].

### Data analysis methods

**Fig 7:**
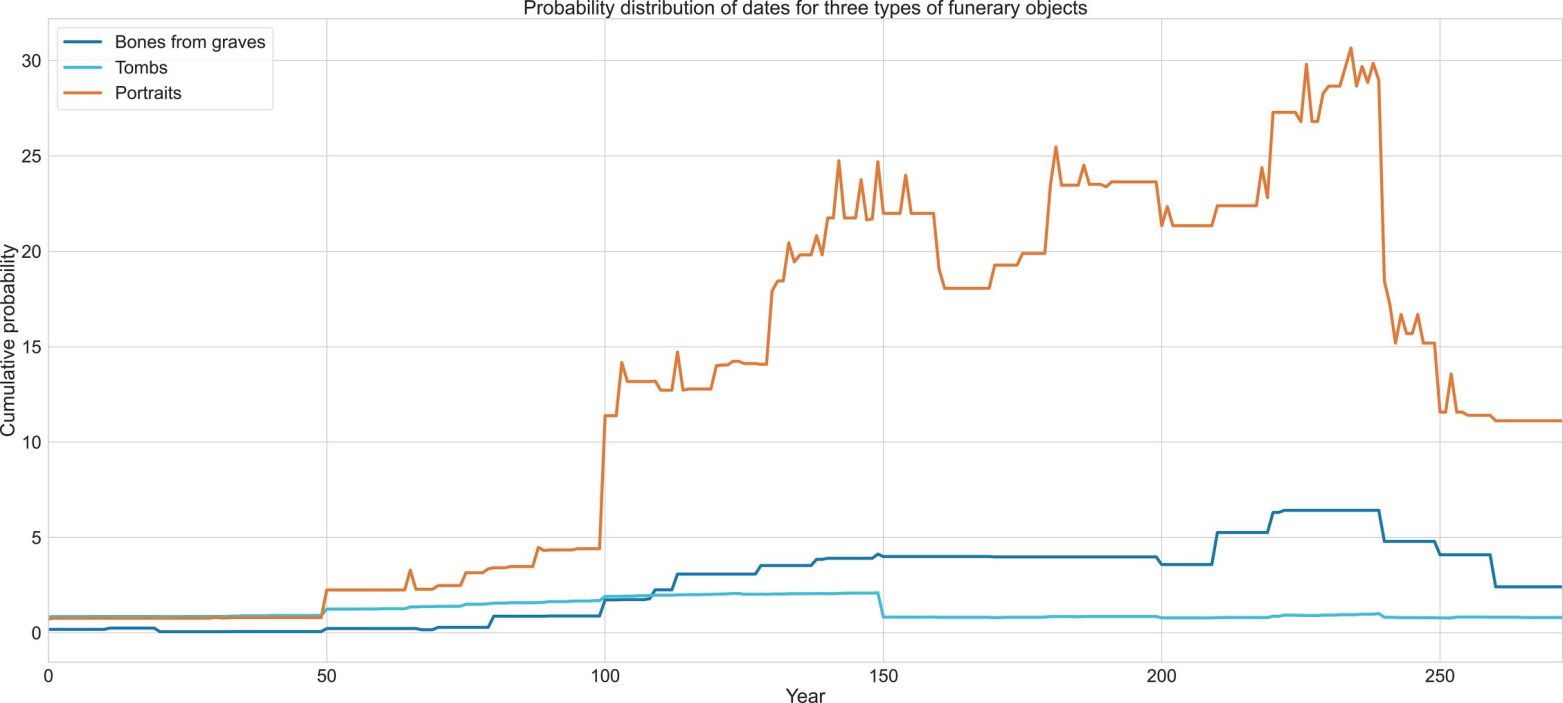
Probability distribution of portraits depicting men, women and children. Graph © authors.

The overall conservatism displayed in the style of the portraits is today to our advantage, since the portrait corpus allows us to explore even the slightest changes and continuities, and this now allows us to model the elite’s funerary behavior over centuries through the tight dating we can impose on the material’s development. We also situated the portraits within the local, regional and global context Palmyrene society was in across 300 years [13, 93] (Fig 7).

Furthermore, to contextualize the data and compare it with other types of evidence, we studied the overall development of the tombs in Palmyra, their date of construction and capacities [125]. In our calculations, we also took into consideration that the portraits depict deceased people and therefore they in fact express the living society with one generation’s delay.

**Fig 8:**
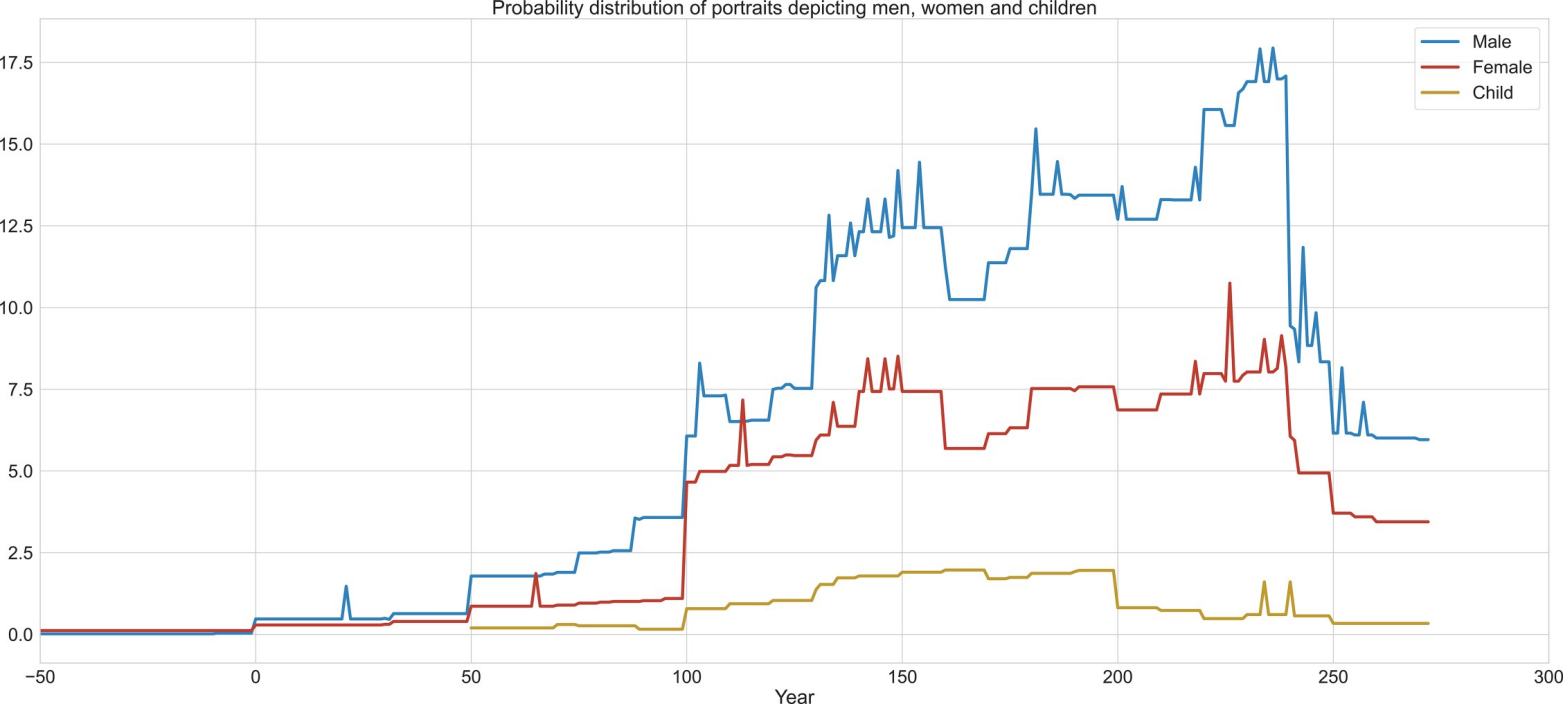
Probability distribution of dates for three types of funerary objects (portraits, tombs, bones from graves). Graph © authors.

We used a standard cumulative probability method (aoristic methods) to map out the frequency of portraits and other funerary evidence from the site [for full analysis, see 125]. The analysis scripts were developed in Python [126] using its main Data Science libraries [pandas: 127, matplotlib: 128, seaborn: 129] and are available via Jupyter Notebook [130] in Supplementary Information B [131]. Objects were dated down to chronological intervals, which were then summed up revealing the general trends of object frequency over time. The resulting probability curve (Figs 7 and 8) depicts a summed probability of all the objects that have been produced at the time. The method gives more weight to more tightly dated objects, thus preserving the chronological uncertainty of the dating, while avoiding the biases inherent to simpler methods such as taking the average age or binning the dates in pre-defined periods. In addition, the certainty of each date (the start and the end date of the dating interval) has been quantified and the robustness of the emerging patters checked for different levels of chronological certainty among the objects [for more details see: 125; for the full analysis script see 131].

In addition to the portraits, we quantify the funerary structures–the number of tombs and their estimated capacity. This data comes with several caveats. First, only 50 graves display in-situ funerary portraits, and most portraits have only become known after they–at unknown points in time–have been removed from their original location. Thus, each data type is treated as independent from each other, as the majority of portraits cannot be directly associated with a particular burial or even tomb. Second, while the tombs known from Palmyra could have housed substantially more burials than the ones counted through the funerary objects, it is not possible to say whether the often monumental structures were in fact ever meant to have been entirely filled. Their sizes could also partly have been exaggerated for representative reasons. The particular patterns identified in the additional datasets are discussed in detail in [125].

## Results

Through the modeling of the portraits on the basis of the chronological studies done within the project it is now possible to trace the flows and fluctuations in the production of these and tie the various phases of production intensity into the overall archaeological and historical development narratives of Palmyra (Fig 7).

The overall modeling of the production intensity of the portraits held together with the development of the funerary structures show complex processes that may be related to several societal factors. The overall developments can be used as evidence to detect general trends in rises and declines in the production of portraits. They may give certain levels of insight into overall patterns of death rates within the elite, at least in terms of the minimum numbers. However, they predominantly show the general developments in terms of economic and societal trends among the city elites. The funerary sphere was one of the main ways of displaying one’s wealth and prestige, and the number of portraits demonstrates what proportion of the society could afford such expensive displays.

There is a clear rise in production in the first and early second centuries CE, a slowdown and plateau in the middle of the second century CE. A dramatic drop beginning approximately 160 CE and continuing until 175 CE is detected. A further plateau is seen between 175 and 220 CE, another peak between 220 and 240 CE and lastly a dramatic drop in the period 240–272 CE (Fig 8).

Both portraits’ and the burials’ curves exhibit this general pattern, whereas the tomb data agrees only until the mid-second century after which the tomb numbers and capacity drop dramatically.

We have investigated the last pattern in detail [125] and concluded that it is most likely caused by the city being saturated with burial capacity by the mid-second century CE. The appearance of the phenomenon of concessions, where part of the tomb capacity was sold to another family, further supports this observation.

The significant conclusions, which can be drawn from the most robust curve (given the number of data points), i.e. the portraits, are the following: the rapid rise in the production of the portraits mirrors a general upswing in the economy of Palmyra, as a result of the political and military stability in the region, in turn leading to an increase in accumulated wealth. This increase in wealth was put towards, among other things, portrait production, which was a way of representing the city’s elite to their surrounding world, and indicates a rapid growth of Palmyrene society in general, expansion and division of labor forces, including an increase in specialization of labor, such as craftsmanship. The middle of the second century CE indicates a stable period, coinciding more or less with the Hadrianic and Antonine periods, which we know were overall periods of political stability in the Roman Near East.

It is noticeable that the immediate impact of the Antonine Plague around 160 CE can be traced in the portrait production, which dips dramatically from 160 CE until approximately 175 CE. About 175 CE the production reached a new plateau lasting for more than a generation, before increasing once more in the early third century, a time in which we know that Palmyra was politically, militarily, and trade-wise a strong player in the Roman East. The epidemic seems to have impacted Palmyrene society for about a generation according to the production curve. Around 240 CE there is a strong decrease in production of the portraits, which may be a sign of the downturn for the city, or at least a decrease of resources that could be dedicated to the funerary sphere. The Parthian invasion of Dura-Europos in 239 CE, a border town east of Palmyra, and a second invasion in 240 CE of Hatra further East, seem to have been significant for the city of Palmyra. These two events led to a mobilization of the Palmyrene army financed and led by the elite. This mobilization can be read in the decrease in production of portraits, which would have been affected dramatically, since economic resources would have been diverted to other areas of the city’s life, such as the army. Furthermore, the male population, including the elite male members, would have been involved in military activities and for large parts have left the city at least for a while, leaving numerous work-intensive production areas without sufficient labor.

After the destabilizing events around 239 CE, Palmyrene society does not seem to have recovered, and although production of the portraits continued, underlining the strong local tradition, they were never produced on the same scale as earlier. It is particularly interesting since this period being regarded as the “golden age” of Palmyra and associated with increased political, economic, religious and financial power yielded by Palmyra’s elites [1, 37, 132–137]. The conflicts therefore seem to have had a significant impact on Palmyrene society’s resilience in general and negatively influenced the ability to regenerate–even when viewed over several decades. In the second century CE a dramatic drop in the tomb capacities is noted, underlining a saturation of the surrounding landscape with monumental graves above ground and underground graves. Since many tombs could hold several hundreds of burials, it is likely that the tomb capacity was plentiful at this point in time, and that financial resources were directed towards other projects, such as decoration projects and other central urban building projects. Through the written evidence we know that Palmyra’s elite was reorganized into four main tribes [135]. Whether this in fact had a direct impact on the organization of the funerary sphere, we cannot detect in the funerary corpus or the structure of the graves. This might imply that the reorganization, as suggested by some scholars [133], was in fact only an administrative one and not one which had an impact on the cultural and social structure of Palmyrene society. The Palmyrene society seems to have adhered to local values that can be traced across the about 300 years in which the portraits were produced [3, 63].

Gender divisions stayed consistent over time with an approximate 70–30 male-female ratio, and children were consistently represented as well, but most often together with deceased parents [51, 136, 137] (Fig 7). The portraits of the deceased elite members can be divided as follows: 1643 male portraits, 481 portraits of priests, 1205 of women and 216 of children. 151 portraits are so fragmented that it is not possible to tell the gender of the represented person.

While it is not surprising that fewer females are present in the corpus than males, it is still significant that female portraits constitute 32.5% of the entire body of material [58]. This underlines that elite women in Palmyra were continuously represented in the funerary sphere, not only as wives of elite men, but also in their own rights, a fact that has often been ignored in scholarship. While such trends now can be followed in detail due to the descriptions of the single portraits, it is just as significant what can be concluded about broader trends on the basis of the development of the portraits. It is clear from the material evidence that male portraiture, including dress codes and styles, continuously kept to a more conservative style than the female portraiture [93]. However, hairstyles for example do show Roman imperial and Parthian influences, and through these broader trends circulating in the Roman and Parthian Empires and beyond can be traced [93]. Children were represented in their own rights in a number of cases (42 examples) [e.g. 52, 53, 57], but most often they were depicted together with their deceased parents underlining the importance of showing continuation of the family beyond parental generations (169 examples). The use of language in the graves also reflect an almost exclusive focus on the local language, Palmyrene Aramaic, despite the fact that Palmyra in the public sphere display more bilingual inscriptions than any other city in the Roman Near East. Out of 1189 funerary inscriptions, only 12 are in Greek and 5 in Latin. Palmyrene Aramaic consistently stayed the dominating language in the funerary sphere, underlining the elite’s focus on their own lineages and local traditions. The Greek and Latin inscriptions in the funerary sphere appeared almost exclusively in the later second century CE, hinting at the fact that this was a time of renewed influence from the Roman world [42].

## Discussion

The modeling of the funerary portraits reveals profound societal transformations, while also displaying continuity in the self-representation of the Palmyrene elite. These changing patterns had until now been only partly traceable through scattered epigraphic evidence and through very few and chronologically later written sources, or they had not been known at all. Although the funerary portraits were not used by all segments of the city’s inhabitants, but limited to use within the elite, they give direct insight into the economic and societal patterns within this prominent group of individuals in the city, patterns which in turn can be said to be valid for the rest of Palmyrene society, since elites in Antiquity were the driving forces behind urban economies and inter-urban networks [138, 139]. While it is well known that Palmyra’s urban area expanded in the course of the first and second centuries CE, following a trend that is broadly detectable in the Roman Near East, and that its society flourished in these periods as well, it is now possible for the first time to follow the city’s societal transformation through the funerary portraits and the mapping of the development and capacities of the monumental graves.

The vast amount of well-dated portraits, according to absolute and relative chronologies, combined with the fact that the production of the limestone funerary portraits continued for three centuries, provide us with an entirely new level of detail about an important segment of Palmyrene society, the elite. This allows for tracing both elite phenomena as well as broader developments in the overall make-up of Palmyrene society, addressing questions such as workforce capacity, economic growth as reflected in the production of the portraits and their quality [94, 140], changing fashion trends and use of language over time [3].

Although we are aware that the database does not include all the portraits produced in ancient Palmyra, it represents the current and also the future state of our knowledge regarding Palmyrene’s funerary sphere. Any significant changes to the dating of these portraits in the future are unlikely, since we here present the material known to its fullest extent–most of which is ex-situ. While some new graves might be discovered in the future–although unlikely due to the nature of the destruction of the cultural heritage in Syria over the last ten years during the civil war–these are unlikely to furnish so many new portraits as to contravene our stylistic typologies based on thousands of examples. There are also no techniques or methods which allow for a close dating of, for example, the local limestone, which would be able to redate these portraits in an even higher resolution than the one presented here. This places the current chronology as a benchmark for other chronologies based on different types of data, which, if systematically collected, could shed light on the historical trajectories of Palmyra’s population.

On the other hand, it is not possible to draw direct conclusions about causality from a single dataset. The correlations between historical events presented here are of interest as they may indicate relationship between societal and economic perturbations in the region and the Palmyrene society, but equally they need not to be regarded as proven until more datasets or more advanced modeling techniques can confirm these conclusions. As such, this study serves as a benchmark against which further studies can compare their results. In particular, quantitative methods could be applied to other classes of archaeological material–pottery, inscriptions, metals, or coinage.

With these caveats in mind, the modeling of the archaeological material makes it possible for us to make significant contributions to discussions of historical developments already known from epigraphic or later written sources. The two most important findings are the drops in portrait production connected to the Antonine Plague and the one around 240 CE, which we connect to the Parthian invasion of Dura-Europos in 239 CE–an indicator for political and military instability. It is in particular relevant to note two key findings of this study. First, not all historical events had the same impact on the city–some military conflicts leave a trace in the data, while others do not. The impact of the Antonine Plague is visible through a sudden drop in the production of the portraits; likewise is the possibly devastating impact of the Parthian invasion of Dura-Europos, also visible through a sudden drop in the production of funerary portraits, which never experienced another surge. Second, Palmyrene society stood under the influence of such events for a longer period of time than usually thought. It seems to have taken Palmyra about a generation’s time to regenerate from the impact of the Antonine Plague. The time span of a generation also indicates that the instability brought by the epidemic would, apart from having affected society through a sudden rise in deaths, also have had a destabilizing influence on the society’s economy, from which it obviously took some time to recover. In turn, this indicates that there would have been less trade activity going on in this period and that Palmyrene society might have been more isolated than before and after the epidemic. The last dramatic drop in the production of the portraits, which we connect with the Parthian invasions of Dura-Europos in 239 CE and of Hatra in 240 CE, shows that Palmyra never recovered from the instability caused by this invasion and the continuing political and military instability. Financial resources would have been directed to other realms, such as the mobilization of the Palmyrene army. We know from historical sources that the Palmyrene Kingdom rose in this period, but it ended with the uprising by Palmyra’s short-term ruler Zenobia against the Romans and the devastating sacks of the city in 272 and 273 CE. So the last dramatic drop in the production of the funerary portraits may also indicate an overall decline in the society’s ability to regenerate economic resilience. We know that Palmyra was in crisis in the third century CE due to a number of factors–the growing instability of the Roman Empire and in its border regions in general, which had an impact on the trade, the core income factor, of the city. Furthermore, the Palmyrenes had been trying to expand their territory, with some luck, and this would also have meant that resources, including monetary resources, would have been pulled out of the society and put into the military upkeep. This might also have meant that graves and funerary traditions could not have been upkept as they had been before.

### Future directions

The work showcased here is extendable to other parts of the ancient world. While not many other societies will hold such an amount of chronologically well-dated material, like the funerary portraits from Palmyra, there are immense perspectives in undertaking such studies. For example, contrasting this with material from Asia Minor, Rome, Italy in general and Greece might throw entirely new light on the archaeological record. This paper has sought to demonstrate how such archaeological data may inform us about developments known or unknown from historical sources and to show in which ways a local society might have been affected by events such as epidemics and political and military instability. The study presented here has wide implications for future modeling of scenarios pertaining to the past in general and can be widened to other categories of material culture produced over long time spans.

While we might not be able to go to a microscopic level and be informed about, for example, climate change in this period through the flows and fluctuations in the portraits, we are still able to consider aspects such as how fast society recovered from an epidemic or from military instability in the region. The material presented here opens new avenues for studying the long-term trends in societal responses to crises, and these trends in societal changes can, in turn, be matched against other sources of data, for example, climate-related data.

The current portrait corpus is likely to involve a significant proportion of Palmyrene portraits produced in Antiquity, and there is very little chance that a new collection or a new undisturbed grave will be discovered that would be large enough to significantly change the trends detected in almost 4000 portraits. Therefore, it is necessary to view the developments in the light of other groups of material in order to gain as much information as possible. This includes epigraphic material, literary sources, as well as knowledge about Palmyra’s urban development and historical developments of the site and region in general. While such material is limited and scattered, it can be used with confidence when viewed in connection with the statistically solid corpus of the funerary portraits. In this way, such scattered data can now be applied and evaluated on entirely different grounds than earlier, which in turn will give new insights into how archaeological data may be used in historical contexts in future studies.

## Conclusions

Palmyra is a well-known Roman-period middle-sized urban city displaying a bulk of archaeological material, including the recently compiled, locally produced funerary portraits set up as commemoration memorials for local elite members in monumental graves. These portraits and funerary monuments have been considered in this paper and add significantly to our knowledge about the site’s and its society’s development across almost 300 years. While Palmyra has been the subject of research for more than 100 years, the consideration given here specifically to the 3704 funerary portraits, which were produced continuously throughout the Roman period, allows for entirely new insights into the city’s archaeology and history. The results highlighted in this paper show how archaeological material can be mapped against historical events and how the material indeed also may reveal events and processes which the historical sources have not left traces of. While not always allowing for firm conclusions about the events and processes behind flows and fluctuations in the production of funerary portraits, the material brings to the forefront the unleashed potential of archaeological baseline projects, which despite being entirely based on, in this case, classical archaeological and art-historical methodologies, indeed allow for firm integration into a wider historical narrative about the city’s development over centuries.

The work undertaken within the framework of the Palmyra Portrait Project is basic humanities research. However, since the work has been done in extreme detail, it has been possible to map and model the portraits with wide implications for our understanding of Palmyrene society’s development, societal changes, and economic, health, and military fluctuations, which in turn inform us about the political climate of the time–not only locally but on a much broader scale transcending imperial borders. Thus the results presented here call for further corpora of archaeological material to be studied in this way and discussed together with already published groups of materials such as the current one. The data and the contextualized results presented here show how archaeological data from one site might impact widely beyond the site itself and how we may begin to study the past in a significantly different way.

This article contains supporting information [131, 141, 142].

## Supporting information

S1 Table Dated Palmyrene objects. (DOCX)

S2 Table Colledge, typology*. (DOCX)

S3 Table Ingholt, typology*. (DOCX)

S4 Table Palmyra portrait project, typology. (DOCX)

S1 Data (CSV)

S2 Data (XLSX)

S3 Data (CSV)

S4 Data (XLSX)

S5 Data (PDF)

S1 File (DOCX)

## References

[1] Ingholt H. Studier over palmyrensk skulptur. Copenhagen: C. A. Reitzels Forlag; 1928.

[2] Colledge MAR. The art of Palmyra. London: Thames and Hudson; 1976.

[3] Raja R. Family matters. Family constellations in Palmyrene funerary sculpture. In: Bøggild Johannsen K, Petersen JH, editors. Family lives. Aspects of life and death in ancient families. Copenhagen: Museum Tusculanum Press; 2019. pp. 245–270.

[4] Albertson F. Typology, attribution, and identity in Palmyran funerary portraiture. In: Kropp A, Raja R, editors. The world of Palmyra. Copenhagen: The Royal Danish Academy of Sciences and Letters; 2016. pp. 150–165.

[5] Romanowska I, Brughmans T, Bes P, Carrignon S, Egelund L, Lichtenberger A, et al. A study of the centuries-long reliance on local ceramics in Jerash through full quantification and simulation. J Archaeol Method Theory. 2021;481. 10.1007/s10816-021-09510-0.

[6] Romanowska I, Lichtenberger A, Raja R. Trends in ceramic assemblages from the Northwest Quarter of Gerasa/Jerash, Jordan. J Archaeol Sci Rep. 2021;34:102778. 10.1016/j.jasrep.2020.102778.

[7] Carrignon S, Brughmans T, Romanowska I. Tableware trade in the Roman East: Exploring cultural and economic transmission with agent-based modelling and approximate Bayesian computation. PLoS ONE. 2020;15(11):e0240414, 11.2020. doi: 10.1371/journal.pone.0240414 33237902 PMC7688115

[8] Binford SR, Binford L. New perspectives in archaeology. Chicago: Aldine Press; 1968.

[9] Chippindale C. Review of "Processual archaeology and the radical critique". Curr Anthropol. 1987;28(4): 501–538.

[10] Raja R, Bobou O, Yon J-B. The Palmyrene funerary portraits. Turnhout: Brepols. Forthcoming.

[11] Raja R. Palmyrene funerary portraits. Collection histories and current research. In: Aruz J, editor. Palmyra. Mirage in the desert. New York: Metropolitan Museum; 2018. pp. 100–109.

[12] Raja R. Urbanizing the desert. Investigating the diversity of urban networks through the images of deceased Palmyrenes. In: Raja R, Sindbæk SM, editors. Urban network evolutions. Towards a high-definition archaeology. Aarhus: Aarhus University Press; 2018. pp. 75–80.

[13] Raja R. Funerary portraiture in Palmyra. Portrait habit at a crossroads or a signifier of local identity? In: Blömer M, Raja R, editors. Funerary portraiture in Greater Roman Syria. Turnhout: Brepols; 2019. pp. 95–110.

[14] Raja R. It stays in the family. Palmyrene priestly representations and their constellations. In: Krag S, Raja R, editors. Women, children and the family in Palmyra. Copenhagen: The Royal Academy of Sciences and Letters; 2019. pp. 95–156.

[15] Raja R. Palmyra Portrait Project_full bibliography_2013–2021_MARCH.pdf. Figshare [deposited 2020 May 8]. Available from: https://figshare.com/articles/dataset/Palmyra_Portrait_Project_full_bibliography_March_2021/14259707.

[16] Raja R. The history and current situation of world heritage sites in Syria. The case of Palmyra. In: Almqvist K, Belfrage L, editors. Cultural heritage at risk. The role of museums in war and conflict. Stockholm: Axel and Margaret Ax:son Johnson Foundation; 2016. pp. 27–47.

[17] Raja R. Illegal trade and export of cultural goods. The case of the Palmyrene funerary portraiture. In Chahin D, Lindblom I, editors. Fighting the looting of Syria’s cultural heritage. Report from the Sofia conference 16 September. Initiatives to stop illicit antiquities trade financing the Syrian conflict. Awareness rising. Oslo: Norwegian Institute for Cultural Heritage Research; 2016. pp. 11–12.

[18] Raja R. The Palmyra Collection. Copenhagen: Ny Carlsberg Glyptotek; 2019.

[19] Raja R. Portrait habit in Palmyra. In: Nielsen AM, Raja R, editors. The road to Palmyra. Copenhagen: Ny Carlsberg Glyptotek; 2019. pp. 137–154.

[20] Sartre-Fauriat A. The discovery and reception of Palmyra. In: Nielsen AM, Raja R, editors. The road to Palmyra. Copenhagen: Ny Carlsberg Glyptotek; 2019. pp. 65–76.

[21] Smith AMI. Roman Palmyra. Identity, community, and state formation. Oxford: Oxford University Press; 2013.

[22] Sommer M. Palmyra. Biographie einer verlorenen Stadt. Darmstadt: Philipp von Zabern; 2017.

[23] Anfinset N, Meyer JC. The hinterland of Palmyra. Antiquity Project Gallery. 2010;84(324). Available from:http://antiquity.ac.uk/projgall/anfinset324/ http://www.antiquity.ac.uk/projgall/anfinset324/.

[24] Anfinset N, Hesse KJ. Palmyrena. Palmyra and the surrounding territory. Joint Syrian-Norwegian project. Surface survey north of Palmyra, April and May 2011. Preliminary report, prehistorical periods. Bergen: Department of Archaeology, History, Cultural Studies and the History of Religions, University of Bergen; 2013. Available from: http://hdl.handle.net/1956/10476.

[25] Meyer JC, Seland EH, Anfinset N, editors. Palmyrena. City, hinterland and caravan trade between Orient and Occident. Proceedings of the conference held in Athens, December 1–3, 2012. Oxford: Archaeopress Archaeology; 2016.

[26] Gawlikowski M. The making of a city. In: Nielsen AM, Raja R, editors. The road to Palmyra. Copenhagen: Ny Carlsberg Glyptotek; 2019. pp. 78–90.

[27] Raja R. Urban development and regional identity in the eastern Roman provinces, 50 BC-AD 250. Aphrodisias, Ephesos, Athens, Gerasa. Copenhagen: Museum Tusculanum Press; 2012.

[28] Lichtenberger A, Raja R. Late Hellenistic and Roman Antioch on the Chrysorrhoas, also called Gerasa. A reappreciation of the evidence in the light of the findings of the Danish-German Jerash Northwest Quarter Project (2011–2017). In. Lichtenberger A, Raja R, editors. Hellenistic and Roman Gerasa. The archaeology and history of a Decapolis city. Turnhout: Brepols; 2020. pp. 7–54.

[29] Hanson JW. An urban geography of the Roman world. 100 BC to AD 300. Oxford: Archaeopress; 2016.

[30] Yon J-B. Les notables de Palmyre. Beirut: Institut Français du Proche Orient; 2002.

[31] Yon J-B. Palmyra and its elites. In: Nielsen AM, Raja R, editors. The road to Palmyra. Copenhagen: Ny Carlsberg Glyptotek; 2019. pp. 92–108.

[32] Sartre M. D’Alexandre à Zénobie. Histoire du Levant antique. IVe siècle avant J.-C.-IIIe siècle après J.-C. Paris: Fayard; 2001. doi: 10.1046/j.1365-2850.2001.00405.x

[33] Sartre-Fauriat A, Sartre M. Palmyre. La cité des caravanes. Paris: Découvertes Gallimard; 2008.

[34] Seland EH. Palmyrene long-distance trade. Land, river, and maritime routes in the first three centuries CE. In: Walter NM, Ito-Adler JP, editors. Long-distance trade, culture, and society. Cambridge: Cambridge Institutes Press; 2015. pp. 101–131.

[35] Seland EH. Ships of the desert and ships of the sea. Palmyra in the world trade of the first-third centuries CE. Wiesbaden: Harrassowitz Verlag; 2016.

[36] Schörle K. Palmyrene merchant networks and economic integration in competitive markets. In: Seland EH, Teigen HF, editors. Sinews of empire. Networks in the Roman Near East and beyond. Oxford: Oxbow Books; 2017. pp. 147–154.

[37] Edwell PM. Between Rome and Persia. The middle Euphrates, Mesopotamia and Palmyra under Roman control. London & New York, Routledge; 2008.

[38] Hartmann U. Das palmyrenische Teilreich. In: Johne K-P, editor. Die Zeit der Soldatenkaiser. Krise und Transformation des Römischen Reiches im 3. Jahrhundert. Berlin: De Gruyter; 2008. pp. 343–378.

[39] Hartmann U. What was it like to be a Palmyrene in the age of crisis? Changing Palmyrene identities in the third century AD. In: Kropp A, Raja R, editors. The world of Palmyra. Copenhagen: The Royal Danish Academy of Sciences and Letters; 2016. pp. 53–69.

[40] Intagliata EE. Palmyra after Zenobia AD 273–750. An archaeological and historical reappraisal. Oxford: Oxbow Books; 2018.

[41] Intagliata EE. The city that would not fall. Palmyra in late antique and early Islamic times. In: Nielsen AM, Raja R, editors. The road to Palmyra. Copenhagen: Ny Carlsberg Glyptotek; 2019. pp. 233–250.

[42] Raja R. Pearl of the desert. A history of Palmyra (New York: Oxford University Press). Forthcoming.

[43] Raja R. Negotiating social and cultural interaction through priesthoods. The iconography of priesthood in Palmyra. In: Hoffman-Salz J, editor. The Middle East as middle ground? Cultural interaction in the ancient Middle East revisited. Holzhausen: Vienna; 2021, pp. 129–146.

[44] Gawlikowski M. Monuments funéraires de Palmyre. Warsaw: Panstwowe Wydawnictwo Naukowe, Éditions scientifiques de Pologne; 1970.

[45] Schmidt-Colinet A. Das Tempelgrab Nr. 35 in Palmyra. Studien zur Palmyrenischen Grabarchitektur und ihrer Ausstattung. Mainz am Rhein: Philipp von Zabern; 1992.

[46] Henning A. Die Turmgräber von Palmyra. Eine lokale Bauform im kaiserzeitlichen Syrien als Ausdruck kultureller Identität. Rahden: Verlag Marie Leidorf; 2013.

[47] Henning A. The tower tombs of Palmyra. Chronology, architecture and decoration. Studia Palmyrenskie. 2013;12: 159–176.

[48] Raja R, Sørensen AH. The “Beauty of Palmyra” and Qasr Abjad (Palmyra). New discoveries in the archive of Harald Ingholt. JRA. 2015;28(1): 439–450.

[49] Henning A. Houses of eternity. The funerary monuments of Palmyra. In: Nielsen AM, Raja R, editors. The road to Palmyra. Copenhagen: Ny Carlsberg Glyptotek; 2019. pp. 156–172.

[50] Raja R. Men of the desert or men of the world? Revisiting the iconography of Palmyrene men and their camels. In: Mæhle IB, Ravnå P, Seland EH, editors. Methods and models in ancient history. Essays in honor of Jørgen Christian Meyer. Athens: Norwegian Institute at Athens; 2020. pp. 129–150.

[51] Krag S, Raja R. Unveiling female hairstyles. Markers of age, social roles, and status in the funerary sculpture from Palmyra. Zeitschrift für Orientarchäologie. 2018;11: 242–277.

[52] Krag S, Raja R. Representations of women and children in Palmyrene banqueting reliefs and sarcophagus scenes. Zeitschrift für Orient-Archäologie. 2017;10: 196–227.

[53] Krag S, Raja R. Representations of women and children in Palmyrene funerary loculus reliefs, loculus stelae and wall paintings. Zeitschrift für Orient-Archäologie. 2016;9: 134–178.

[54] Blömer M, Raja R. Shifting the paradigms. Towards a new agenda in the study of the funerary portraiture of Greater Roman Syria. In: Blömer M, Raja R, editors. Funerary portraiture in Greater Roman Syria. Turnhout: Brepols; 2019. pp. 5–26.

[55] Raja R. Palmyrene funerary portraits in context. Portrait habit between local traditions and imperial trends. In: Fejfer J, Moltesen M, Rathje A, editors. Traditions. Transmission of culture in the ancient world. Copenhagen: Museum Tusculanum Press; 2015. pp. 329–361.

[56] Krag S. Palmyrene funerary buildings and family burial patterns. In: Krag S, Raja R, editors. Women, children and the family in Palmyra. Copenhagen: The Royal Academy of Sciences and Letters; 2019. pp. 38–66.

[57] Krag S, Raja R. Families in Palmyra. The evidence from the first three centuries CE. In: Krag S, Raja R, editors. Women, children and the family in Palmyra. Copenhagen: The Royal Academy of Sciences and Letters; 2019. pp. 7–18.

[58] Seyrig H. Note sur les plus anciennes sculptures palmyréniennes. Berytus; 1936;3: 137–40.

[59] Henning A. The representation of matrimony in the tower tombs of Palmyra. In: Krag S, Raja R, editors. Women, children and the family in Palmyra. Copenhagen: The Royal Academy of Sciences and Letters; 2019. pp. 19–37.

[60] Schmidt-Colinet A. Zwei Neufunde Palmyrenischer Sarkophage. In: Koch G, editor. Symposium des Sarkophag-Corpus, Marburg 2001. Mainz am Rhein: Philipp von Zabern; 2007. pp. 271–278; pls. 284–290.

[61] Kropp AJM, Raja R. The Palmyra Portrait Project. In: Álvarez JM, Nogales T, Rodà I, editors. Proceedings of the XVIIIth international congress of classical archaeology. Vol. II. Centre and Periphery. Mérida: Museo Nacional de Arte Romano; 2015. pp. 1223–1226.

[62] Parlasca K. Syrische Grabreliefs hellenistischer und römischer Zeit. Mainz am Rhein: Philipp von Zabern; 1982.

[63] Raja R. Stacking aesthetics in the Syrian desert. Displaying Palmyrene sculpture in the public and funerary sphere. In: Draycott C, Raja R, Welch K, Wootton WT, editors. Visual histories. Visual remains and histories of the classical world. Papers in honour of R.R.R. Smith. Turnhout: Brepols; 2019. pp. 281–298.

[64] Raja R. Going individual. Roman period portraiture in classical archaeology. In: Lichtenberger A, Raja R, editors. The diversity of classical archaeology. Turnhout: Brepols; 2017. pp. 271–286.

[65] De Jong L. The archaeology of death in Roman Syria. Burial, commemoration and empire. Cambridge: Cambridge University Press; 2017.

[66] Eger C, Mackensen M, editors. Death and burial in the Near East from Roman to Islamic times. Research in Syria, Lebanon, Jordan and Egypt. Wiesbaden: Reichert Verlag; 2018.

[67] Will E. La tour funéraire de la Syrie et les monuments apparentés. Syria. 1949;26(3–4): 258–312.

[68] Will E. La tour funéraire de Palmyre. Syria. 1949;26(1–2): 87–116.

[69] Amy R, Seyrig H. Recherches dans la nécropole de Palmyre. Syria. 1936;17(3): 229–266.

[70] Abdul-Hak S. L’hypogée de Taai à Palmyre. Ann Archeol Syrie. 1952;2: 193–251.

[71] Sadurska A, Bounni A. Les Sculptures Funéraires de Palmyre. Rome: Giorgio Bretschneider; 1994.

[72] Saliby N, Parlasca K. L’hypogée de Sassan fils de Malê à Palmyre. Damaszener Mitteilungen. 1992;6: 267–292.

[73] Schmidt-Colinet A. Das Tempelgrab Nr. 36 in Palmyra. Studie zur Palmyrenischen Grabarchitektur und ihrer Ausstattung, 2 vols. Mainz am Rhein: Philipp von Zabern; 1992.

[74] Makowski CK. Recherches sur le banquet miniaturisé dans l’art funéraire de Palmyre. Studia Palmyreńskie. 1985;8: 119–130.

[75] Parlasca K. Beobachtungen zur palmyrenischen Grabarchittektur. Damaszener Mitteilungen. 1989;4: 181–190.

[76] Cussini E. Legal formulary from Syriac documents and Palmyrene inscriptions. An overview. In: Démare-Lafont S, Lemaire A, editors. Trois millénaires de formulaires juridiques. Geneva: Libraire Droz S.A.; 2010. pp. 337–355.

[77] Ingholt H. Five dated tombs from Palmyra. Berytus. 1935;2: 58–120.

[78] Cantineau J. Inventaire des inscriptions de Palmyra, VIII. Le dépot des Antiquités. Beirut: Imprimerie Catholique; 1932.

[79] Higuchi T, Saito K. Tomb F. Tomb of BWLH and BWRP. Southeast necropolis Palmyra, Syria. Nara: Research Center for Silk Roadology; 2001.

[80] Saito K. Palmyrene burial practices from funerary goods. In: Cussini E, editor. A journey to Palmyra. Collected essays to remember Delbert R. Hillers. Leiden: Brill; 2005. pp. 150–165.

[81] Al-Hariri K. The tomb of ‘Aqraban. Studia Palmyreńskie. 2013;12: 149–157.

[82] Musée Impérial Ottomann. Antiquités Himyarites et Palmyréniennes. Catalogue sommaire Constantinople: Musée Impérial Ottomann; 1895.

[83] Palaz Erdemir H. Mystery of the funerary reliefs of Palmyra (Tadmor) in the desert of Syria. Turkish Studies. 2013;8(7): 507–518.

[84] Pasinli A. Istanbul Archaeological Museums. Istanbul: A Turizm Yayinlari; 2001.

[85] Dentzer-Feydy J, Teixidor J. Les antiquités de Palmyra au Musée du Louvre. Paris: Èditions de la Réunion des Musées Nationaux; 1993.

[86] Masson M. Two Palmyrene stelae from the Merv Oasis. East and West. 1967;17:239.47.

[87] Trendall AD. The shellal mosaic and other classical antiquities in the Australian War Memorial Canberra. Canberra: Australian War Memorial; 1973. pp. 28–29, pl. V.

[88] Krag S. Funerary representations of Palmyrene women. From the first century BC to the third century AD. Turnhout: Brepols; 2018.

[89] Parlasca K. La sculpture grecque et la sculpture d’époque romaine Impériale en Syrie. In: Dentzer J-M, Orthmann W, editors. Archéologie et histoire de la Syrie II: La Syrie de l’époque achéménide à l’avènement de l’Islam. Saarbrücken: Saarbrücker Druckerei und Verlag; 1989. pp. 537–556, 546–547, fig. 203.

[90] Cleveland Museum of Art. Procession of Nobles, AD 100–150, Syria, Palmyra. Available from: https://www.clevelandart.org/art/1970.15#.

[91] Heyn M, Raja R, editors. Individualizing the dead. Attributes in Palmyrene funerary sculpture. Turnhout: Brepols; 2021.

[92] Bobou O, Kristensen N, Jensen JV, Raja R, Thomsen RR. Studies on Palmyrene sculpture. A commented translation of Harald Ingholt’s 1928 Studier over palmyrensk skulptur. Turnhout: Brepols; 2021. doi: 10.1001/jamanetworkopen.2020.26874

[93] Raja R. Powerful images of the deceased. Palmyrene funerary portrait culture between local, Greek and Roman representations. In: Boschung D, Queyrel F, editors. Bilder der Macht. Das griechische Porträt und seine Verwendung in der antiken Welt. Paderborn: Wilhelm Fink; 2017. pp. 319–348.

[94] Steding J. Carvers and customers. The production economy of limestone loculus reliefs from Roman Palmyra, 1st to 3rd century AD. Turnhout: Brepols. Forthcoming.

[95] Parlasca K. Das Verhältnis der palmyrenischen Grabplastik zur römischen Porträtkunst. Mitteilungen des Deutschen Archäologischen Instituts, Römische Abteilung. 1985;92: 343–356.

[96] Parlasca K. Römische Elements in der Grabkunst Palmyras. Petra and the Caravan Cities: Proceedings of the Symposium Organised at Petra in September 1985. Amman: Departmnet of Antiquities; 1990.

[97] Higuchi T, Izumi T, editors. Tombs A and C, Southeast Necropolis, Palmyra, Syria. Surveyed in 1990–92, Publication of Research Center for Silk Roadology, 1. Nara: Research Center for Silk Roadology; 1994.

[98] Miyashita S. The vessels in Palmyrene banquet scenes. Tomb BWLH and BWRP amd Tomb TYBL. In: Meyer JC, Seland EH, Anfinset N, editors. Palmyrena. City, hinterland and caravan trade between Orient and Occident. Proceedings of the conference held in Athens, December 1–3, 2012. Oxford: Archaeopress; 2016. pp. 131–146.

[99] Will E. Le relief de la tour de Khitôt et le banquet funéraire à Palmyre. Syria; 1951;28(1–2): 70–100.

[100] Seyrig H. Le repas des morts et le “banquet funèbre” à Palmyre. Ann Archeol Syrie. 1951;1: 32–40.

[101] Audley-Miller L. The banquet in Palmyrene funerary contexts. In: Draycott CM, Stamatopoulou M, editors. Dining and death. Interdisciplinary perspectives on the ‘funerary banquet’ in ancient art, burial and belief. Colloquia Antiqua, 16. Leuven: Peeters; 2016. pp. 553–590.

[102] Ringsborg S. Children’s portraits from Palmyra. In: Raja R, editor. Palmyra. Pearl of the desert. Aarhus: SUN-Tryk; 2017. pp. 67–76.

[103] Heyn M. Gesture at Dura-Europos. A new interpretation of the so-called ‘scène énigmatique’. In: Kaizer T, editor. Religion, society and culture at Dura-Europos. Cambridge: Cambridge University Press; 2016. pp. 105–115.

[104] Long T. Facing the evidence. How to approach the portraits. In: Kropp A, Raja R, editors. The world of Palmyra. Copenhagen: The Royal Danish Academy of Sciences and Letters; 2016. pp. 135–149.

[105] Heyn MK, Raja R. Male dress habits in Roman period Palmyra. In: Cifarelli M., editor. Fashioned selves. Dress and identity in antiquity. Oxford: Oxbow Books; 2019. pp. 41–51.

[106] Heyn MK. Gesture and identity in the funerary art of Palmyra. AJA. 2010;114(4): 631–661.

[107] Davies G. The body language of Palmyra and Rome. In: Long T, Sørensen AH, editors. Positions and professions in Palmyra. Copenhagen: The Royal Danish Academy of Sciences and Letters; 2017. pp. 20–35.

[108] Sokolowski L. Portraying the literacy of Palmyra. The evidence of funerary sculpture and its interpretation. Études et travaux. 2014;27: 376–403.

[109] Bobou O. Plants in Palmyrene funerary iconography of adults. In: Heyn MK, Raja R, editors. Individualizing the dead. Attributes in Palmyrene funerary sculpture. Turnhout: Brepols; 2021. pp. 31–50.

[110] Soltan A. Ikonografia meharystów palmyrénskich. Studia Palmyreńskie. 1969;3: 5–46.

[111] Raja R. Representations of priests in Palmyra. Methodological considerations on the meaning of the representation of priesthood in the funerary sculpture from Roman period Palmyra. Religion in the Roman Empire. 2016;2(1): 125–146.

[112] Raja R. Between fashion phenomena and status symbols. Contextualising the dress of the so-called "former priests" of Palmyra. In: Brøns C, Nosch M-L, editors. Textiles and cult in the Mediterranean area in the 1st millennium BC. Oxford: Oxbow Books; 2017. pp. 209–229.

[113] Raja R. Priesthood in Palmyra. Public office or social status? In: Raja R, editor. Palmyra. Pearl of the desert. Aarhus: SUN-Tryk; 2017. pp. 77–85.

[114] Raja R. You can leave your hat on. Priestly representations from Palmyra. Between virual genre, religious importance and social status. In: Gordon RL, Petridou G, Rüpke J, editors. Beyond priesthood, religious entrepreneurs and innovators in the imperial era. Berlin: de Gruyter; 2017. pp. 417–442.

[115] Raja R. "The matter of the Palmyrene ’modius’. Remarks on the history of research of the terminology of the Palmyrene priestly hat. Religion in the Roman Empire. 2018;4(2): 237–59.

[116] Raja R. To be or not to be depicted as a priest in Palmyra. A matter of representational spheres and societal values. In: Long T, Sørensen AH, editors. Positions and professions. Copenhagen: The Roal Academy of Sciences and Letters; 2017. pp. 115–130.

[117] Raja R. Individualising Palmyrene priests through priestly attributes. Abstract from Attributes in Palmyrene art and sculpture, Aarhus, Denmark, 19 June 2018.

[118] Curtis VS. The Parthian haute-couture at Palmyra. In: Long T, Sørensen AH, editors. Positions and professions in Palmyra. Copenhagen: The Royal Danish Academy of Sciences and Letters; 2017. pp. 52–67.

[119] Heyn MK. Sacerdotal activities and Parthian dress in Roman Palmyra. In: Colburn CS, Heyn MK, editors. Reading a dynamic canvas. Adornment in the ancient Mediterranean world. Cambridge: Cambridge Scholars Publishing; 2008. pp. 170–193.

[120] Krag S. Women in Palmyra. In: Raja R, editor. Palmyra. Pearl of the desert. Aarhus: SUN-Tryk; 2017. pp. 57–66.

[121] Krag S. Changing identities, changing positions. Jewellery in Palmyrene female portraits. In: Long T, Sørensen AH, editors. Positions and professions in Palmyra, Palmyrene Studies, 2. Copenhagen: The Royal Danish Academy of Sciences and Letters; 2017. pp. 36–51.

[122] Thomsen RR. Unlocking a mystery. The keys in Palmyrene funerary portraiture. In: Heyn MK, Raja R, editors. Individualizing the dead. Attributes in Palmyrene funerary sculpture. Turnhout: Brepols; 2021. pp. 51–62.

[123] Raja R, Yon J-B. Palmyrene funerary sculptural representations with Greek, Latin and bilingual inscriptions. Zeitschrift für Orientarchäologie. Forthcoming.

[124] Albertson F. The ‘fringed’ mangle and its relation to gender in Palmyrene funerary sculpture. In: Heyn MK, Raja R, editors. Individualizing the dead. Attributes in Palmyrene funerary sculpture. Turnhout: Brepols; 2021. pp. 13–30.

[125] Romanowska I, Bobou O, Raja R. Reconstructing the social, economic and demographic trends of Palmyra’s elite from funerary data. Journal of Archaeological Science. 2021; 105432. doi: 10.1016/j.jas.2021.105432

[126] Rossum G van, Drake FL. The Python language reference. Release 3.0.1 [Repr.]. Hampton, NH: Python Software Foundation; 2010.

[127] Reback J, McKinney W, Jbrockmendel, Bossche JVD, Augspurger T, Cloud P, et al. pandas-dev/pandas: Pandas 1.0.3. Zenodo; 2020. doi: 10.5281/ZENODO.3715232

[128] Hunter JD. Matplotlib: A 2D Graphics Environment. Comput Sci Eng. 2007;9: 90–95. doi: 10.1109/MCSE.2007.55

[129] Waskom M, Botvinnik O, O’Kane D, Hobson P, Lukauskas S, Gemperline DC, et al. Mwaskom/Seaborn: V0.8.1 (September 2017). Zenodo; 2017. doi: 10.5281/ZENODO.883859

[130] Kluyver T, Ragan-Kelley B, Pérez F, Granger B, Matthias M, Frederic J, et al. Jupyter Notebooks—a publishing format for reproducible computational workflows. Stand Alone. 2016; 87–90. doi: 10.3233/978-1-61499-649-1-87

[131] Romanowska I. Palmyra’s funerary data analysis (Version 1.0.0). Zenodo; 2021. 10.5281/zenodo.4674905.

[132] Edwell PM. Palmyra between Rome and the Parthians. In: Nielsen AM, Raja R, editors. The road to Palmyra. Copenhagen, Ny Carlsberg Glyptotek; 2019. pp. 109–126.

[133] Hartmann U. Das palmyrenische Teilreich. Stuttgart, Franz Steiner Verlag; 2001.

[134] Sommer M. Palmyra. A history. Abingdon, Oxen, Routledge; 2018.

[135] Kaizer T. The religious life of Palmyra. A study of the social patterns of worship in the Roman period. Stuttgart: Franz Steiner Verlag; 2002.

[136] Raja R. Representations of the so-called "former priests" in Palmyrene funerary art. A methodological contribution and commentary. Topoi Orient Occident. 2017;21(1): 51–81.

[137] Krag S, Raja R, editors. Women, children and the family in Palmyra. Copenhagen: The Royal Academy of Sciences and Letters; 2019.

[138] Scheidel W, Friesen SJ. The size of the economy and the distribution of income in the Roman empire. JRS. 2009;99: 61–91.

[139] Raja R. Networking beyond death. Priests and their family networks explored through the funerary sculpture. In: Seland EH, Teigen HF, editors. Sinews of empire. Networks in the Roman Near East and beyond. Oxford: Oxbow Books; 2017. pp. 121–136.

[140] Raja R, Steding J. Production economy in Roman Syria. New views on old stones. In: Raja R, Steding J, editors. Production economy in Greater Roman Syria. Trade networks and production processes. Turnhout: Brepols; 2021.

[141] Raja R. Palmyra portrait typology and chronology (version 1.0.0) [Dataset]. Zenodo; 2021. Available from: 10.5281/zenodo.4736592.

[142] Raja R. Funerary data from Palmyra, Syria collated by the Palmyra Portrait Project (Version 1.0.0) [Dataset]. Zenodo; 2021. Available from: 10.5281/zenodo.4669962.

