# Supplementary material for: Three hundred years of Palmyrene history. Unlocking archaeological data for studying past societal transformations
Source: PLoS One. 2021 Nov 3;16(11):e0256081. doi: 10.1371/journal.pone.0256081 (PMC8565770; doi:10.1371/journal.pone.0256081)
Supplement: S1 Table — (DOCX) [file pone.0256081.s001.docx]

**Dated Palmyrene objects**

| **Date** | **Current location** | **Inv. no.** | **PAT** | ***CIS*** | **Other publication** |
| --- | --- | --- | --- | --- | --- |
| ad 21/22 | Palmyra Museum, Palmyra, Syria | CD 11/77  Votive relief |  |  | Gawlikowski 2017, 245–46, cat. 15. |
| ad 40 | In situ, Tower tomb no. 44, Tower of Kithot, Palmyra, Syria. | Unknown inv. no.  Foundation relief | 0464 | 4115 |  |
| ad 55 | British Museum, London, United Kingdom | BM 125206  Votive relief | 0318 | 3972 |  |
| ad 64 | In situ, Temple of Allat, the column of Šalamallat, Palmyra, Syria. | Unknown inv. no.  Column | 0312 | 3966 | Yon 2012, 134–35, cat. 124. |
| ad 65/66 | Ny Carlsberg Glyptotek, Copenhagen, Denmark | IN 2816  Loculus relief | 0001 |  | Raja 2019, 66–67, cat. 1. |
| ad 73 | In situ, Tower tomb no. 194, Tower of Ogeilu, Palmyra, Syria (Henning 2013 reports that it is not in place anymore, so possibly lost) | Unknown inv. no.  Foundation relief | 0549 | 4193 |  |
| ad 85 | Oxford, Ashmolean Museum | C2-9  Altar | 0324 | 3978 |  |
| ad 89/(1)89 | Palmyra Museum, Palmyra, Syria | CD 36  Loculus relief |  |  | Gawlikowski 1974, 75–76, cat. 147, pl. 8. |
| ad 96 | Ny Carlsberg Glyptotek, Copenhagen, Denmark | IN 1057  Loculus relief | 0712 | 4354 | Raja 2019, 68–69, cat. 2. |
| ad 103 | In situ, Tower tomb no. 13, Tower of Elahbel, Palmyra, Syria. | Foundation relief |  | 4134 | Yon 2012, 319–20, cat. 407. |
| ad 103/104 | In situ, hypogeum of Bôlḥâ, north exedra, sec. 18, loc. 1, Palmyra, Syria. | Unknown inv. no.  Loculus relief | 1871 |  |  |
| ad 108 | National Museum of Damascus, Damascus, Syria | Unknown inv. no.  lintel | 2784 |  |  |
| ad 113/114 | British Museum, London, United Kingdom | BM 125695  Loculus relief | 0732 | 4374 |  |
| ad 113/114 | Ny Carlsberg Glyptotek, Copenhagen, Denmark | IN 1079  Loculus relief | 0733 | 4374bis | Raja 2019, 70–71, cat. 3. |
| ad 113/213 | Palais d'Azem, Damascus, Syria (last known location) | Unknown inv. no.  Votive relief | 0320 | 3974 |  |
| ad 114 | State Hermitage Museum, St. Petersburg, Russia | ДВ-4177  Stele | 0481 | 4129 |  |
| ad 115 | Palmyra Museum, Palmyra, Syria | CD 70/65  Altar | 1929 |  |  |
| ad 123 | The Royal Ontario Museum, Toronto, Canada | 953X94.1  Loculus relief | 1751 |  |  |
| ad 125/126 | Ny Carlsberg Glyptotek, Copenhagen, Denmark | IN 1155  Loculus relief relief | 0603 | 4247 | Raja 2019, 72–73, cat. 4. |
| ad 128 | Damascus, Directorate-General of Antiquities and Museums, Syria | Unknown inv. no.  Loculus relief |  |  | unpublished |
| ad 128 | In situ, tower tomb no. N206, Palmyra, Syria. | Unknown inv. no.  Sarcophagus box |  |  | al-As'ad 2013, 16, fig. 1. |
| ad 130 | Musée National de Beyrouth, Beirut, Lebanon | Unknown inv. no.  Stele | 0155 |  |  |
| ad 133-134 | Ny Carlsberg Glyptotek, Copenhagen, Denmark | IN 1049  Loculus relief | 0638 | 4281 | Raja 2019, 74–75, cat. 5. |
| ad 133/134 | Unknown, Damascus, Syria | Unknown inv. no.  Loculus relief | 0602 | 4246 |  |
| ad 134/135 | American University of Beirut, Archaeological Museum, Beirut, Lebanon | 2740  Loculus relief | 0604 | 4248 |  |
| ad 138 | Palmyra Museum, Palmyra, Syria | B 2687/9088  Loculus relief |  |  | Yon 2013, 343, cat. 30. |
| ad 138 | Robert Mouawad Private Museum, Beirut, Lebanon | 0149  Votive relief | 2625 |  |  |
| ad 138/139 | Musée Art & Histoire, Brussels, Belgium | A 1620  Loculus relief | 0613 | 4257 |  |
| ad 139 | Private collection, unknown | Unknown inv. no.  Loculus relief |  |  | Yon 2013, 347, cat. 60. |
| AD 140/141 | Private collection, Deratiyeh | Unknown inv. no.  Sarcophagus box |  |  | unpublished |
| ad 141 | Saint Louis Art Museum, Saint Louis, Missouri, USA | 24:1960  Loculus relief | 0922 | 4561 |  |
| ad 142/143 | In situ, hypogeum of the three brothers, south exedra, west wall, Palmyra, Syria. | Unknown inv. no.  Full sarcophagus | 2776 |  |  |
| ad 145 | Musei Vaticani, Rome, Vatican city | VII 61/ MV 15029  Loculus relief | 0605 | 4249 |  |
| ad 146 | The Metropolitan Museum of Art, New York, New York, USA | 02.29.3  Loculus relief | 0614 | 4258 |  |
| ad 146/147 | Ny Carlsberg Glyptotek, Copenhagen, Denmark | IN 1159  Banqueting relief | 0818 | 4458 | Raja 2019, 76–79, cat. 6. |
| ad 146-147 | Ny Carlsberg Glyptotek, Copenhagen, Denmark | IN 1160  Banqueting relief | 0818 | 4458 bis | Raja 2019, 76–79, cat. 6. |
| ad 148 | National Museum of Damascus, Damascus, Syria | 15028  Sarcophagus box | 0005 |  | Cussini 2017, 88. |
| ad 149 | Ny Carlsberg Glyptotek, Copenhagen, Denmark | IN 2794  Loculus relief | 1752 |  | Raja 2019, 80–81, cat. 7. |
| ad 150 | Syriac Catholic Patriarchate, Beirut, Lebanon | Unknown inv. no.  Loculus relief | 0625 | 4268 |  |
| ad 150 | American University of Beirut, Archaeological Museum, Beirut, Lebanon | 2739  Loculus relief | 0610 | 4254 |  |
| ad 151 | American University of Beirut, Archaeological Museum, Beirut, Lebanon | 2738  Loculus relief | 0721 | 4363 |  |
| ad 151 | Robert Mouawad Private Museum, Beirut, Lebanon | 0101  Loculus relief | 0813 | 4453 |  |
| ad 152 | Palmyra Museum, Palmyra, Syria | A 1234  Votive relief | 1568 |  |  |
| ad 154 | National Museum of Damascus, Damascus, Syria | C 2842  Votive relief | 1670 |  |  |
| ad 154/155 | State Hermitage Museum, St Petersburg, Russia | ДВ-8841  Loculus relief | 0939 | 4578 |  |
| ad 154/155 | Unknown location | Unknown inv. no.  Loculus relief | 0022 |  |  |
| ad 155 | Musée du Louvre, Paris, France | AO 2201  Loculus relief | 0607 | 4251 |  |
| ad 157 | İstanbul Arkeoloji Müzesi, Istanbul, Turkey | 3840  Loculus relief | 0977 | 4616 |  |
| ad 160 | Nationalmuseet, Copenhagen, Denmark | 14489  Loculus relief | 0009.2 |  |  |
| ad 160/161 | In situ, hypogeum of Bôlḥâ, north exedra, sec. 30, loc. 2, Palmyra, Syria. | Unknown inv. no.  Loculus relief | 1880 |  |  |
| ad 161 | Hotel Des Ventes de Neuilly-sur-Seine, Paris, France (last known location) | Unknown inv. no.  Loculus relief | 0923 | 4562 |  |
| ad 161– 163 | Ny Carlsberg Glyptotek, Copenhagen, Denmark | IN 2775  Loculus relief | 1633 |  | Raja 2019, 82–85, cat. 8. |
| ad 161– 163 | Iris and B. Gerald Cantor Center for Visual Arts at Stanford University, California, USA | 17200  Loculus relief | 1652 |  | Albertson 2012, 254–58. |
| ad 164 | Private collection, Lebanon | Unknown inv. no.  Loculus relief |  |  | Abousamra 2015, 229–33, no. iv, figs. 11–13; Krag 2018, 263–64, cat. 363. |
| ad 165/166 | Damascus DGAM | Unknown inv. no.  Loculus relief relief |  |  | Unpublished |
| ad 165/166 | Palmyra Museum, Palmyra, Syria | CD 122/60  Loculus relief relief | 1956 |  |  |
| ad 169 | National Museum of Damascus, Damascus, Syria | Unknown inv. no.  Loculus relief | 0611 | 4255 |  |
| ad 172 | The Metropolitan Museum of Art, New York, New York, USA | 02.29.6  Loculus relief relief | 0618 | 4261bis |  |
| ad 175/176 | Palmyra Museum, Palmyra, Syria | A 125  Loculus relief |  |  | Cantineau 1932, 76, cat. no. 114 |
| ad 176 | Musée du Louvre, Paris, France | AO 2200  Loculus relief relief | 0606 | 4250 |  |
| ad 177 | Palmyra Museum, Palmyra, Syria | 1988/7113  Loculus relief | 2677 |  |  |
| ad 181 | American University of Beirut, Archaeological Museum, Beirut, Lebanon | 2733  Loculus relief | 0612 | 4256 |  |
| ad 181 | The Metropolitan Museum of Art, New York, New York, USA | 02.29.4  Loculus relief | 0620 | 4263 |  |
| ad 181 | Ny Carlsberg Glyptotek, Copenhagen, Denmark | IN 2774  Loculus relief | 0888 | 4527 | Raja 2019, 86–87, cat. 9. |
| ad 181/182 | Palmyra Museum, Palmyra, Syria | B 1948/7040  Loculus relief | 1025 |  | As'ad and Gawlikowski 1997, 44, cat. 58, fig. 58 |
| ad 184 | British Museum, London, United Kingdom | BM 125150  Loculus relief | 0868 | 4507 |  |
| ad 186 | Yale University Art Gallery, New Haven, Connecticut, USA | 1954.30.3  Loculus relief | 0910 | 4549 |  |
| ad 186/187 | Private collection of the military commander, Palmyra, Syria | Unknown inv. no.  Loculus relief | 1749 |  |  |
| ad 188 | Palmyra Museum, Palmyra, Syria | A 910/10  Full sarcophagus |  |  | Yon 2013, 340, cat. 11a-f |
| ad 189 | State Hermitage Museum, St Petersburg, Russia | ДВ-8840  Loculus relief | 0649 | 4292 |  |
| ad 189 | Palmyra Museum, Palmyra, Syria | CD 56/73  Loculus relief | 1649 |  | Krag 2018, 273, cat. 396. |
| ad 190 | Palmyra Museum, Palmyra, Syria | CD 37/74  Relief |  |  | Gawlikowski 2017, 245, cat. 14 |
| ad 191 | National Museum of Damascus, Damascus, Syria | Unknown inv. no.  Votive relief |  |  | Schlumberger 1951, 156, cat. 39 |
| ad 192 | Palmyra Museum, Palmyra, Syria | CD 133  Loculus relief | 1971 |  |  |
| ad 199 | National Museum of Damascus, Damascus, Syria | Unknown inv. no.  Votive relief | 1667 |  |  |
| ad 201/202 | Baron Poche collection, Aleppo, Syria (last known location) | Unknown inv. no.  Loculus relief | 0651 | 4294 |  |
| ad 204 | Gołuchów Castle Museum, Poznań, Poland | Unknown inv. no.  Loculus relief | 0799 | 4439 |  |
| ad 211 | The Fralin Museum of Art at the University of Virginia, Charlottesville, Virginia, USA | 2001.16.2  Loculus relief | 0887 | 4526 |  |
| ad 211 | Muzeum Narodowe w Warszawie, Warsaw, Poland | 199576  Loculus relief | 0830 | 4469 | Krag 2018, 325, cat. 579 |
| ad 215 | Musée de Baalbek, Baalbek, Lebanon (last known location) | Unknown inv. no.  Loculus relief | 0915 | 4554 |  |
| ad 218/219 | Istanbul Arkeoloji Müzesi, Istanbul, Turkey | 3783/O.M.242  Loculus relief | 0663 | 4306 |  |
| ad 225 | National Museum of Damascus, Damascus, Syria | C2118 or 4480  Votive relief | 2757 |  |  |
| ad 226/227 | Skulpturensammlung, Staatliche Kunstsammlungen Dresden, Dresden, Germany | Hm 025  Loculus relief | 0657 | 4300 |  |
| ad 226/227 | National Museum of Damascus, Damascus, Syria | C 9  Loculus relief | 0600 | 4244 |  |
| ad 233/234 | Kulturhistorisk museum, Universitetet i Oslo, Oslo, Norway | C42231  Loculus relief | 0665 | 4308 |  |
| ad 233/234 | National Museum of Damascus, Damascus, Syria | C 22  Loculus relief | 0599 | 4243 |  |
| ad 234/235 | Vorderasiatisches Museum, Berlin, Germany | VA 2660  Loculus relief | 0658 | 4301 |  |
| ad 236/237 | American University of Beirut, Archaeological Museum, Beirut, Lebanon | 32.25  Loculus relief | 0019 |  |  |
| ad 238/239 | Palmyra Museum, Palmyra, Syria | CD 114  Loculus relief | 1964 |  | Krag 2018, 358, cat. 731. |
| ad 239 | Palmyra Museum, Palmyra, Syria | 1795/6644, 1769/6645  Full sarcophagus | 1526-1535 |  | al-As'ad and Gawlikowski 1997, 46–47, cat. 65, fig. 65. |
| ad 240 | Bibliothéque Nationale et Universitaire, Strasbourg, France | Unknown inv. no.  Votive relief | 0383 | 4037 |  |
| ad 240 | Palmyra Museum, Palmyra, Syria | A 1175  Votive relief | 1908 |  |  |
| ad 240/241 | Vorderasiatisches Museum, Berlin, Germany | VA 16754  Loculus relief | 0659 | 4302 |  |
| ad 240/241 | Kulturhistorisk museum, Universitetet i Oslo, Oslo, Norway | C42237  Loculus relief | 0664 | 4307 |  |
| ad 241/242 | Henri Marcopoli collection, Aleppo, Syria (last known location) | Unknown inv. no.  Loculus relief | 0662 | 4305 |  |
| ad 241/242 | Freer Gallery of Art, Washington DC, USA | 08.236  Loculus relief | 0821 | 4460 |  |
| ad 243/244 | Palmyra Museum, Palmyra, Syria | A 1174  Votive relief | 1909 |  |  |
| ad 243/247 | In situ, tower tomb of Iamliku (no. 51), Palmyra, Syria. | Unknown inv. no.  Sarcophagus lid |  |  | Gawlikowski and al-As’ad 1997, 32–33, cat. 17, pl. 5.2. |
| ad 246/247 | Musée National de Beyrouth, Beirut, Lebanon | Unknown inv. no.  Loculus relief | 1750 |  |  |
| ad 252/253 | Robert Mouawad Private Museum, Beirut, Lebanon | Unknown inv. no.  Loculus relief | 2630 |  |  |
| ad 252/253 | Palmyra Museum, Palmyra, Syria | A 1223/6310  Loculus relief |  |  | Yon 2013, 344, cat. 40. |
| ad 258 | Christie's, New York, New York, USA | Unknown inv. no.  Votive relief |  |  | Yon 2013, 360, cat. 157. |
| ad 263 | National Museum of Damascus, Damascus, Syria | C 3841  Votive relief | 1724 |  |  |

**Bibliography**

CIS *Corpus Inscriptionum Semiticarum.* Paris: E Reipublicae Typographeo.

PAT D. R. Hillers and E. Cussini. 1996. *Palmyrene Aramaic Texts* (Baltimore – London: The John Hopkins University Press).

Abousamra, G. 2015. ‘Palmyrene Inscriptions on Seven Reliefs’, *Semitica* 57: pp. 217–42.

al-As’ad, K. and M. Gawlikowski 1997. *The Inscriptions in the Museum of Palmyra. A catalogue*. (Palmyra, Warsaw: Oficyna wydawnicza).

al-As’ad, W. 2013. ‘Some tombs recently excavated at Palmyra’, *Studia Palmyreńskie* 12: pp. 15–24.

Albertson, F. C. 2012. ‘The ‘Date’ on Two Dated Palmyran Funerary Reliefs’, *Zeitschrift für Orient-Archäologie* 5: 250–70.

Cantineau, J. 1932. *Inventaire des inscriptions de Palmyre. VIII. Le dépot des antiquités* (Beyrouth: Publications du Musée National Syrien de Damas).

Cussini, E. 2017. ‘The Pious Butcher and the Physicians. Palmyrene Professions in Context’, in A. H. Sørensen and T. Long (eds), *Positions and Professions in Palmyra. Palmyrenske Studier* 2. Scientia Danica, Series H, Humanistica 4:9 (Copenhagen: Royal Danish Academy of Sciences and Letters): pp. 84–96.

Gawlikowski, M. 1974. *Recueil d’inscriptions palmyréniennes provenant de fouilles syriennes et polonaises récentes à Palmyre* (Paris: Imprimerie Nationale).

Gawlikowski, M. 2017. *Le sanctuaire d’Allat à Palmyre* (Warsaw: Wydawnictwo Uniwersytetu Warszawskiego).

Krag, S. 2018. *Funerary Representations of Palmyrene Women. From the First Century BC to the Third Century AD* (Turnhout: Brepols).

Raja, R. 2019. *The Palmyra Collection. Ny Carlsberg Glyptotek* (Copenhagen: Ny Carlsberg Glyptotek).

Schlumberger, D. 1951. *La Palmyrene du Nord-Ouest* (Paris: Librairie orientaliste Paul Geuthner).

Yon, J.-B. 2012. *Inscriptions grecques et latines de la Syrie 17.1. Palmyre* (Beirut: Institut Français du Proche Orient).

— 2013. ‘Palmyrene epigraphy after PAT, 1996-2011’, *Studia Palmyreńskie* 12: 333–67.
