# Supplementary material for: Three hundred years of Palmyrene history. Unlocking archaeological data for studying past societal transformations
Source: PLoS One. 2021 Nov 3;16(11):e0256081. doi: 10.1371/journal.pone.0256081 (PMC8565770; doi:10.1371/journal.pone.0256081)
Supplement: S2 Table — (DOCX) [file pone.0256081.s002.docx]

**COLLEDGE, TYPOLOGY***

Colledge, M. 1976. *The Art of Palmyra* (London: Thames and Hudson).

*The table relates the defining, overall characteristics of group I–III given by Colledge (1976, 245–64).

| DATE | GENDER | HEADDRESS | HAIRSTYLE | EYEBROWS | EYES | BEARD (yes/no) | CLOTHES | JEWELLERY | ATTRIBUTES | POSE |
| --- | --- | --- | --- | --- | --- | --- | --- | --- | --- | --- |
|  | Male | | | | | | | | | |
| 50–150 |  | – | – | Curving grooves | Irises and pupils indicated by concentric, incised circles | No | Tunic  Himation, semi-circular folds  Chlamys | – | Schedula (r. and l. hand)  Leaf (l. hand)  Sword (l. hand)  Bowl (l. hand)  Bird (l. hand) | Arms resting against the chest |
| 150–200 |  | Occasionally a wreath | Greater length and thickness of the hair | Plastically rendered | Irises indicated by concentric, incised circles and pupils indicated by punch holes  Left blank | Both men with beards and without beards appear | Tunic – occasionally with *clavus/clavi*  Himation, semi-circular to loose and naturalistic folds | – | Schedula (l. hand)  Leaf (l. hand) | – |
| 200–273 |  | Occasionally a wreath | Hair varies from short to abundant | Plastically rendered  Curving grooves | Irises indicated by concentric, incised circles  Irises indicated by concentric, incised circles and pupils indicated by punch holes  Left blank | Both men with beards and without beards appear  Beards – often rendered by short strokes | Tunic – occasionally with *clavus/clavi*  Himation, random to semi-circular folds | – | Himation (l. hand)  Schedula (l. hand) | – |
|  | Female | | | | | | | | |  |
| 50–150 |  | Veil, vertical folds | Two shoulder locks  Occasionally locks of hair on the forehead and cheeks | Curving grooves | Irises indicated by concentric, incised circles  Irises and pupils indicated by concentric, incised circles  Left blank | No | Tunic, long sleeved  Himation, semi-circular folds | Earrings, series of small hoops  Earrings, in the shape of bunches of grapes  Earrings, horizontal bars with two or three round pendants  Brooches, trapezoidal | Spindle and distaff (l. hand) | Arms resting against the chest, hands close and more or less opposite each other |
| 150–200 |  | Veil, naturalistic folds | ‘Melon’ hairstyle  One shoulder lock over the right shoulder  Occasionally two shoulder locks | Curving grooves  Curving ridges | Irises indicated by concentric, incised circles  Irises indicated by concentric, incised circles and pupils indicated by punch holes  Left blank | No | Tunic, short sleeved | Earrings, in the shape of bunches of grapes  Earrings, horizontal bars with two or three round pendants  Earrings, dumbbell-shaped  Brooches, trapezoidal, circular, or trapezoidal | Spindle and distaff (l. hand)  Veil (r. and l. hand)  Child (l. hand) | Right (occasionally left) hand raised to the height of the shoulder or neck  Right (occasionally left) hand resting on left cheek |
| 200–273 |  | Veil, naturalistic folds  Turban and headband occasionally abandoned for an embroidered head-cloth | ‘Melon’ hairstyle  Often locks of hair on the forehead and cheeks  Occasionally shoulder locks | – | Irises indicated by concentric, incised circles  Irises indicated by concentric, incised circles and pupils indicated by punch holes  Left blank | No | Tunic, long sleeved – often with embroidery or trimmings | Head ornaments  Earrings, dumbbell-shaped  Brooches, circular – occasionally trapezoidal or polygonal  Bracelets, twisted and beaded  Bracelets with a bell | Veil (r. and l. hand) | Left (occasionally right) hand raised to the height of the shoulder or neck  Left (occasionally right) hand resting on left cheek |
|  | Priests | | | | | | | | |  |
| 50–150 |  | Priestly hat | – | Curving grooves | Irises and pupils indicated by concentric, incised circles | No | Tunic  Himation, semi-circular folds  Chlamys | – | Schedula (l. hand)  Leaf (l. hand)  Bowl (l. hand)  Cup (l. hand)  Alabastron (r. hand) | Arms resting against the chest |
| 150–200 |  | Priestly hat | – | Plastically rendered | Irises indicated by concentric, incised circles and pupils indicated by punch holes  Left blank | No | Tunic – occasionally with *clavus/clavi*  Himation, semi-circular to loose and naturalistic folds | – | Schedula (l. hand)  Leaf (l. hand)  Bowl (l. hand)  Alabastron (r. hand) | – |
| 200–273 |  | Priestly hat | – | Plastically rendered  Curving grooves | Irises indicated by concentric, incised circles  Irises indicated by concentric, incised circles and pupils indicated by punch holes  Left blank | No | Tunic – occasionally with *clavus/clavi*  Himation, random to semi-circular folds | – | Schedula (l. hand)  Leaf (l. hand)  Himation (l. hand) | – |
