# Supplementary material for: Three hundred years of Palmyrene history. Unlocking archaeological data for studying past societal transformations
Source: PLoS One. 2021 Nov 3;16(11):e0256081. doi: 10.1371/journal.pone.0256081 (PMC8565770; doi:10.1371/journal.pone.0256081)
Supplement: S3 Table — (DOCX) [file pone.0256081.s003.docx]

**INGHOLT, TYPOLOGY***

Ingholt, H. 1928. *Studier over Palmyrensk Skulptur* (Copenhagen: C. A. Reitzels Forlag).

*The table relates the defining, overall characteristics of group I–III given by Ingholt (1928, 98–150).

| DATE | GENDER | HEADDRESS | HAIRSTYLE | EYEBROWS | EYES | BEARD (yes/no) | CLOTHES | JEWELLERY | ATTRIBUTES | POSE |
| --- | --- | --- | --- | --- | --- | --- | --- | --- | --- | --- |
|  | Male | | | | | | | | | |
| 50–150 |  | – | – | Curving ridges | Irises indicated by concentric, incised circles | No | Tunic, short sleeved  Himation, semi-circular folds  Himation only covers the left shoulder  Chlamys | – | Schedula (l. hand)  Leaf (l. hand)  Sword (l. hand) | – |
| 150–200 |  | – | – | – | Irises indicated by concentric, incised circles – occasionally pupils indicated by punch holes | Both men with beards and without beards appear | Himation, semi-circular to pointed folds | – | Schedula (r. and l. hand)  Leaf (l. hand)  Whip (r. hand) | Arms resting against the chest  Palm held forward (r. hand) |
| 200–250 |  | – | – | – | – | Both men with beards and without beards appear | Himation, semi-circular to pointed folds | – | Himation (l. hand)  Leaf (l. hand)  Tablet (l. hand)  Schedula (l. hand) | – |
|  | Female | | | | | | | | | |
| 50–150 |  | Veil, diagonal and semi-circular folds  Turban and headband – occasionally abandoned | Two shoulder locks | Curving ridges | Irises and pupils indicated by concentric, incised circles | No | – | Earrings, series of small hoops  Earrings, in the shape of bunches of grapes  Earrings, horizontal bars with two or three round pendants  Brooches, trapezoidal – occasionally with keys | Spindle and distaff (l. hand)  Veil (r. and l. hand)  Child (l. left) | Arms resting against the chest  Right (occasionally left) hand raised to the height of the shoulder or neck  Palm held forward (r. hand) |
| 150–200 |  | Veil, curving folds | ‘Melon’ hairstyle  Two shoulder locks  One shoulder lock over the right shoulder | – | Irises indicated by concentric, incised circles and pupils indicated by punch holes | No | – | Earrings, dumbbell-shaped  Brooches, trapezoidal – occasionally with keys  Brooches, circular – occasionally with keys or threads  Bracelets, twisted | Spindle and distaff (l. hand)  Veil (r. and l. hand)  Child (l. left) | Right (occasionally left) hand raised to the height of the shoulder or neck |
| 200–250 |  | Veil covers the upper right arm – occasionally also the lower right arm  Turban and headband occasionally abandoned for an embroidered head-cloth | Lock under the turban | – | – | No | Tunic, short and long sleeved | Brooches, circular  Bracelets with a bell | Veil (r. and l. hand) | Left (occasionally right) hand raised to the height of the shoulder or neck  Left (occasionally right) hand resting on left cheek |
|  | Priests | | | | | | | | | |
| 50–150 |  | Priestly hat | – | Curving ridges | Irises indicated by concentric, incised circles | No | Tunic, short sleeved  Himation, semi-circular folds  Himation only covers the left shoulder  Chlamys | – | Schedula (l. hand)  Leaf (l. hand)  Bowl (l. hand)  Alabastron (r. hand) | – |
| 150–200 |  | Priestly hat | – | – | Irises indicated by concentric, incised circles – occasionally pupils indicated by punch holes | No | Himation, semi-circular to pointed folds  Chlamys with decorated border | – | Bowl (l. hand)  Alabastron (r. hand) | – |
| 200–250 |  | Priestly hat | – | – | – | No | Himation, rounded folds | – | Schedula (l. hand)  Leaf (l. hand)  Himation (l. hand) | – |
