# Supplementary material for: Three hundred years of Palmyrene history. Unlocking archaeological data for studying past societal transformations
Source: PLoS One. 2021 Nov 3;16(11):e0256081. doi: 10.1371/journal.pone.0256081 (PMC8565770; doi:10.1371/journal.pone.0256081)
Supplement: S4 Table — (DOCX) [file pone.0256081.s004.docx]

**PALMYRA PORTRAIT PROJECT, TYPOLOGY**

| DATE | GENDER/PROFESSION | HEADDRESS | HAIRSTYLE | EYEBROWS | EYES | BEARD (yes/no) | CLOTHES | JEWELLERY | ATTRIBUTES | POSE | INSCRIPTION |
| --- | --- | --- | --- | --- | --- | --- | --- | --- | --- | --- | --- |
|  | Male | | | | | | | | | | |
| 1–100 |  | – | Short hair (Heyn 2016) | Curving grooves (Long 2016) | Irises and pupils indicated by concentric, incised circles (Long 2016) | No (Heyn 2010; 2016) | Tunic, plain (Heyn 2016; Heyn and Raja 2019)  Himation, semi-circular folds (Heyn 2016)  Himation, ‘arm-sling’ type (Heyn 2010; Heyn and Raja 2019) | – | Book-roll (r. and l. hand) (Heyn 2010; 2016; Heyn and Raja 2019)  Leaf (l. hand) (Heyn 2016; Heyn and Raja 2019)  Sword (l. hand) (Heyn 2016)  Whip (l. hand) (Heyn 2016) | – | – |
| 100–135 |  | – | Short hair – comma- to crescent shaped curls arranged in rows (Albertson 2016; Heyn 2016) | Curving grooves (Long 2016) | Irises and pupils indicated by concentric, incised circles (Long 2016) | Both men with beards and without beards appear (Heyn 2010; Albertson 2016) | Tunic, plain (Heyn 2016; Heyn and Raja 2019)  Himation, semi-circular folds (Heyn 2016)  Himation, ‘arm-sling’ type (Heyn 2010; Heyn and Raja 2019) | – | Book-roll (r. and l. hand) (Heyn 2010; 2016; Heyn and Raja 2019)  Leaf (l. hand) (Heyn 2016; Heyn and Raja 2019)  Sword (l. hand) (Heyn 2016)  Whip (l. hand) (Heyn 2016) | – | – |
| 135–160 |  | – | Short hair – comma- to crescent shaped curls arranged in rows (Albertson 2016; Heyn 2016) | Curving grooves (Long 2016) | Irises and pupils indicated by concentric, incised circles (Long 2016) | Both men with beards and without beards appear (Heyn 2010; 2016; Albertson 2016) | Tunic, plain (Heyn 2016; Heyn and Raja 2019)  Himation, semi-circular folds (Heyn 2016)  Himation, ‘arm-sling’ type (Heyn 2010; Heyn and Raja 2019) | – | Book-roll (r. and l. hand) (Heyn 2010; 2016; Heyn and Raja 2019)  Leaf (l. hand) (Heyn 2016; Heyn and Raja 2019)  Sword (l. hand) (Heyn 2016)  Whip (l. hand) (Heyn 2016) | – | Latin (Raja and Yon forthcoming)  Greek (Raja and Yon forthcoming) |
| 160–175 |  | – | Greater length and thickness of the hair (Heyn 2016) | – |  | Both men with beards and without beards appear (Albertson 2016; Heyn 2016) | Tunic, plain (Heyn 2016; Heyn and Raja 2019)  Himation, ‘arm-sling’ type (Heyn 2010; Heyn and Raja 2019) | – | Book-roll (r. and l. hand) (Heyn 2010; Heyn and Raja 2019)  Leaf (l. hand) (Heyn and Raja 2019) | – | Latin (Raja and Yon forthcoming)  Greek (Raja and Yon forthcoming) |
| 175–220 |  | – | Greater length and thickness of the hair (Heyn 2016) | – | Left blank (Long 2016) | Both men with beards and without beards appear (Albertson 2016; Heyn 2016) | Tunic – occasionally with *clavus/clavi* (Heyn and Raja 2019)  Himation, ‘arm-sling’ type (Heyn 2010; Heyn and Raja 2019)  Mantle, fringed (Albertson 2021) | – | Book-roll (r. and l. hand) (Heyn 2010; Heyn and Raja 2019)  Leaf (l. hand) (Heyn and Raja 2019) | – | Latin (Raja and Yon forthcoming)  Greek (Raja and Yon forthcoming) |
| 220–240 |  | – | Occasionally short hair (Heyn 2016) | – | Left blank (Long 2016) | Yes (Heyn 2016) | Tunic – occasionally with *clavus/clavi* (Heyn and Raja 2019)  Himation, ‘arm-sling’ type (Heyn 2010; Heyn and Raja 2019)  Mantle, fringed (Albertson 2021) | – | Book-roll (r. and l. hand) (Heyn 2010; Heyn and Raja 2019)  Leaf (l. hand) (Heyn and Raja 2019)  Himation (l. hand) (Heyn 2016) | – | – |
| 240–272 |  | – | Occasionally short hair (Heyn 2016) | – | Left blank (Long 2016) | Yes (Heyn 2016) | Tunic – occasionally with *clavus/clavi* (Heyn and Raja 2019)  Himation, ‘arm-sling’ type (Heyn 2010; Heyn and Raja 2019) | – | Book-roll (r. and l. hand) (Heyn 2010; Heyn and Raja 2019)  Leaf (l. hand) (Heyn and Raja 2019)  Himation (l. hand) (Heyn 2016) | – | – |
|  | Female | | | | | | | | | | |
| 1–100 |  | Veil, vertical folds (Krag 2018)  Headband, plain or with vertical grooves (Krag 2017b; 2018) | Hair is almost fully covered by headdresses apart from two shoulder locks (Krag 2018; Krag and Raja 2018)  Occasionally locks of hair at the temples (Krag 2018; Krag and Raja 2018) | Curving ridges or grooves (Krag 2018) | Irises and pupils indicated by concentric, incised circles (Long 2016; Krag 2017b; 2018) | No | Tunic, long sleeved and with rounded neckline (Krag 2018)  Himation, semi-circular folds (Krag 2018) | Necklaces, beaded (Krag 2018)  Earrings, series of small hoops (Krag 2018)  Earrings, in the shape of bunches of grapes (Krag 2018)  Earrings, horizontal bars with two or three round pendants (Krag 2018)  Brooches, trapezoidal – occasionally with keys (Krag 2017a; 2017b; 2018; Thomsen 2021) | Spindle and distaff (l. hand) (Heyn 2010; Krag 2017b; 2018) | Frontal – occasionally with subtly turned head (Krag 2018)  Arms resting against the chest (Krag 2018)  Palm held forward (r. hand) (Heyn 2010; Krag 2018) | – |
| 100–135 |  | Veil, vertical folds (Albertson 2016; Krag 2018)  Headband with rectangular panels separated by vertical beaded bands (Krag 2017b; 2018) | Curving locks on both sides of the headdresses at the temples and two shoulder locks (Krag 2018) | Curving ridges or grooves (Krag 2018) | Irises and pupils indicated by concentric, incised circles (Long 2016; Krag 2017b; 2018) | No | Tunic, long sleeved and with rounded neckline (Krag 2018)  Himation, semi-circular folds (Krag 2018) | Necklaces, beaded (Krag 2018)  Earrings, series of small hoops (Krag 2018)  Earrings, in the shape of bunches of grapes (Krag 2018)  Earrings, horizontal bars with two or three round pendants (Krag 2018)  Brooches, trapezoidal – occasionally with keys (Krag 2017a; 2017b; 2018; Thomsen 2021) | Spindle and distaff (l. hand) (Heyn 2010; Albertson 2016; Krag 2017b; 2018) | Frontal – occasionally with subtly turned head (Albertson 2016; Krag 2018)  Arms resting against the chest (Krag 2018)  Palm held forward (r. hand) (Heyn 2010; Krag 2018) | – |
| 135–160 |  | Veil, vertical to naturalistic folds (Albertson 2016; Krag 2018)  Turban, three layers (Krag 2018)  Headband with rectangular panels separated by vertical beaded bands (Krag 2017b; 2018) | Curving locks on both sides of the headdresses at the temples and two shoulder locks (Krag 2018) | Curving ridges or grooves (Krag 2018) | Irises indicated by concentric, incised circles (Krag 2018)  Irises and pupils indicated by concentric, incised circles (Long 2016; Krag 2017b; 2018)  Irises indicated by concentric, incised circles and pupils indicated by punch holes (Krag 2018) | No | Tunic, long sleeved and with rounded neckline (Krag 2018)  Tunic, short sleeved (Krag 2018)  Himation, semi-circular to pointed folds (Krag 2018) | Necklaces, beaded – often with a pendant (Krag 2018)  Necklaces, plaited, twisted, or loop in loop with a pendant (Krag 2018)  Earrings, series of small hoops (Krag 2018)  Earrings, in the shape of bunches of grapes (Krag 2018)  Earrings, horizontal bars with two or three round pendants (Krag 2018)  Earrings, dumbbell-shaped (Krag 2017b; 2018)  Brooches, trapezoidal – occasionally with keys (Krag 2017a; 2017b; 2018 Thomsen 2021) | Spindle and distaff (l. hand) (Heyn 2010; Albertson 2016; Krag 2017b; 2018)  Veil (r. and l. hand) (Krag 2018)  Child (l. hand) (Krag 2018) | Frontal – occasionally with subtly turned head (Albertson 2016; Krag 2018)  Arms resting against the chest (Krag 2018)  Palm held forward (r. hand) (Heyn 2010; 2016; Krag 2018)  Right (occasionally left) hand raised to the height of the shoulder or neck (Heyn 2010; 2016; Krag 2018)  Right (occasionally left) hand resting on left cheek (Heyn 2010; 2016; Krag 2018) | Latin (Raja and Yon forthcoming)  Greek (Raja and Yon forthcoming) |
| 160–175 |  | Veil, naturalistic folds (Krag 2018)  Turban, three layers (Krag 2018)  Headband with rectangular panels separated by vertical beaded bands (Krag 2018) | Curving locks on both sides of the headdresses at the temples and one shoulder lock over the shoulder (Krag 2018)  Occasionally two shoulder locks (Krag 2018)  ‘Faustina’ hairstyle (Albertson 2016; Krag and Raja 2018) | – | Irises indicated by concentric, incised circles (Krag 2018)  Irises and pupils indicated by concentric, incised circles (Krag 2018)  Irises indicated by concentric, incised circles and pupils indicated by punch holes (Krag 2018) | No | Tunic, short sleeved and with v-shaped neckline (Krag 2018)  Himation, pointed folds (Krag 2018) | Necklaces, beaded – often with a pendant (Krag 2018)  Necklaces, plaited, twisted, or loop in loop with a pendant (Krag 2018)  Earrings, in the shape of bunches of grapes (Krag 2018)  Earrings, horizontal bars with two or three round pendants (Krag 2018)  Earrings, dumbbell-shaped (Krag 2017b; 2018)  Brooches, trapezoidal, circular, or polygonal – occasionally with keys (Krag 2017a; 2018) | Spindle and distaff (l. hand) (Krag 2017b; 2018)  Veil (r. and l. hand) (Krag 2018)  Child (l. hand) (Krag 2018) | Frequently turned head (Krag 2018)  Right (occasionally left) hand raised to the height of the shoulder or neck (Heyn 2010; 2016; Krag 2018)  Right (occasionally left) hand resting on left cheek (Heyn 2010; 2016; Krag 2018) | Latin (Raja and Yon forthcoming)  Greek (Raja and Yon forthcoming) |
| 175–220 |  | Veil, naturalistic folds – often with a pleated, scalloped, zigzag, or woolen fringe (Krag 2018)  Veil falls off the shoulders (Krag 2017b)  Turban, three layers (Krag 2018)  Headband with rectangular panels separated by vertical beaded bands (Krag 2018) | Voluminous, curving locks on both sides of the headdresses at the temples and one shoulder lock over the shoulder (Krag 2018)  Occasionally two shoulder locks (Krag 2018)  ‘Faustina’ hairstyle (Albertson 2016; Krag and Raja 2018) | – | Irises indicated by concentric, incised circles (Krag 2018)  Irises and pupils indicated by concentric, incised circles (Krag 2018)  Irises indicated by concentric, incised circles and pupils indicated by punch holes (Krag 2018)  Left blank (Long 2016; Krag 2018) | No | Tunic, short sleeved and with v-shaped neckline – often decorated (Krag 2018)  Himation, pointed folds (Krag 2018)  Mantle, fringed (Albertson 2021) | Necklaces, beaded – often with a pendant (Krag 2018)  Necklaces, plaited, twisted, or loop in loop with a pendant (Krag 2018)  Necklaces, plain with one or two pendants (Krag 2018)  Necklaces with circular and diamond-shaped bezels linked by beaded elements (Krag 2018)  Necklaces, chain with medallion (Krag 2018)  Earrings, in the shape of bunches of grapes (Krag 2018)  Earrings, horizontal bars with two or three round pendants (Krag 2018)  Earrings, dumbbell-shaped (Krag 2017b; 2018)  Brooches, trapezoidal, circular, or polygonal – occasionally with keys (Krag 2017a; 2018)  Bracelets, twisted and beaded (Krag 2018)  Bracelets with a bell (Krag 2018) | Spindle and distaff (l. hand) (Krag 2017b; 2018)  Veil (r. and l. hand) (Krag 2018)  Child (l. hand) (Krag 2018) | Frequently turned head (Krag 2018)  Both right and left hand raised to the height of the shoulder or neck (Heyn 2010; 2016; Krag 2017b; 2018)  Right or left hand resting on the cheek (Heyn 2010; 2016; Krag 2017b; 2018) | Latin (Raja and Yon forthcoming)  Greek (Raja and Yon forthcoming) |
| 220–240 |  | Veil, naturalistic folds – often with a pleated, scalloped, zigzag, or woolen fringe (Krag 2018)  Veil falls off the shoulders (Krag 2017b)  Turban, three layers (Krag 2018)  Headband with rectangular panels separated by vertical beaded bands (Krag 2018) | Voluminous, curving locks on both sides of the headdresses at the temples (Krag 2018)  Occasionally two shoulder locks (Krag 2018)  ‘Faustina’ hairstyle (Albertson 2016; Krag and Raja 2018) | – | Irises indicated by concentric, incised circles (Krag 2018)  Left blank (Long 2016; Krag 2018) | No | Tunic, short sleeved and with v-shaped neckline – often decorated (Krag 2018)  Tunic, fringed and long sleeved (Albertson 2021)  Himation, pointed folds (Krag 2018)  Mantle, fringed (Albertson 2021) | Necklaces, beaded – often with a pendant (Krag 2018)  Necklaces, plaited, twisted, or loop in loop with a pendant (Krag 2018)  Necklaces, plain with one or two pendants (Krag 2018)  Necklaces with circular and diamond-shaped bezels linked by beaded elements (Krag 2018)  Necklaces, chain with medallion (Krag 2018)  Earrings, dumbbell-shaped (Krag 2018)  Brooches, circular or polygonal (Krag 2017a; 2018)  Bracelets, twisted and beaded (Krag 2018)  Bracelets with a bell (Krag 2018) | Veil (r. and l. hand) (Krag 2018) | Frequently turned head (Krag 2018)  Left (occasionally right) hand raised to the height of the shoulder or neck (Heyn 2010; 2016; Krag and Raja 2016; Krag 2017b; 2018)  Left (occasionally right) hand resting on left cheek (Heyn 2010; 2016; Krag 2017b; 2018) | – |
| 240–272 |  | Veil, naturalistic folds – often with a pleated, scalloped, zigzag, or woolen fringe (Krag 2018)  Veil falls off the shoulders (Krag 2017b)  Turban, three layers (Krag 2018)  Headband with rectangular panels separated by vertical beaded bands (Krag 2018) | Voluminous, curving locks on both sides of the headdresses at the temples (Krag 2018)  Occasionally two shoulder locks (Krag 2018)  ‘Faustina’ hairstyle (Albertson 2016; Krag and Raja 2018) | – | Irises indicated by concentric, incised circles (Krag 2018)  Left blank (Long 2016; Krag 2018) | No | Tunic, short sleeved and with v-shaped neckline – often decorated (Krag 2018)  Tunic, fringed and long sleeved (Albertson 2021)  Himation, pointed folds (Krag 2018)  Mantle, fringed (Albertson 2021) | Necklaces, beaded – often with a pendant (Krag 2018)  Necklaces, plaited, twisted, or loop in loop with a pendant (Krag 2018)  Necklaces, plain with one or two pendants (Krag 2018)  Necklaces with circular and diamond-shaped bezels linked by beaded elements (Krag 2018)  Necklaces, chain with medallion (Krag 2018)  Earrings, dumbbell-shaped (Krag 2018)  Brooches, circular or polygonal (Krag 2017a; 2018)  Bracelets, twisted and beaded (Krag 2018)  Bracelets with a bell (Krag 2018) | Veil (r. and l. hand) (Krag 2018) | Frequently turned head (Krag 2018)  Left (occasionally right) hand raised to the height of the shoulder or neck (Heyn 2010; 2016; Krag and Raja 2016; Krag 2017b; 2018)  Left (occasionally right) hand resting on left cheek (Heyn 2010; 2016; Krag 2017b; 2018) | – |
|  | Priests | | | | | | | | | | |
| 1–100 |  | Priestly hat, round, cylindrical, and flat-topped (Raja 2015; 2016; 2017a; 2017c; 2018; 2019; 2021; Heyn and Raja 2019) | Shaved head (Raja 2015; 2016; 2017a; 2017b; 2017c; 2018) | – | – | No (Raja 2016; 2017a; 2017b; 2017c) | Tunic – occasionally embroidered or decorated (Raja 2015; 2016; 2017a; 2017b; 2017c; 2018; 2019; 2021; Heyn and Raja 2019)  Chlamys (Raja 2015; 2016; 2017a; 2017b; 2017c; 2018; 2019; 2021; Heyn and Raja 2019) | Brooch, circular (Raja 2021) | Libation pitcher and incence bowl – often decorated (Raja 2015; 2016; 2017a; 2017b; 2017c; 2018; 2019; 2021; Heyn and Raja 2019) | – | – |
| 100–135 |  | Priestly hat, round, cylindrical, and flat-topped – often with a wreath and a central decoration (Raja 2015; 2016; 2017a; 2017c; 2016; 2018; 2019; 2021; Heyn and Raja 2019) | Shaved head (Raja 2015; 2016; 2017a; 2017b; 2017c; 2018) | – | – | No (Raja 2016; 2017a; 2017b; 2017c) | Tunic – occasionally embroidered or decorated (Raja 2015; 2016; 2017a; 2017b; 2017c; 2018; 2019; 2021; Heyn and Raja 2019)  Chlamys (Raja 2015; 2016; 2017a; 2017b; 2017c; 2018; 2019; 2021; Heyn and Raja 2019) | Brooch, circular (Raja 2021) | Libation pitcher and incence bowl – often decorated (Raja 2015; 2016; 2017a; 2017b; 2017c; 2018; 2019; 2021; Heyn and Raja 2019) | – | – |
| 135–160 |  | Priestly hat, round, cylindrical, and flat-topped – often with a wreath and a central decoration (Raja 2015; 2016; 2017a; 2017c; 2016; 2018; 2019; 2021; Heyn and Raja 2019) | Shaved head (Raja 2015; 2016; 2017a; 2017b; 2017c; 2018) | – | – | No (Raja 2016; 2017a; 2017b; 2017c) | Tunic – occasionally embroidered or decorated (Raja 2015; 2016; 2017a; 2017b; 2017c; 2018; 2019; 2021; Heyn and Raja 2019)  Chlamys (Raja 2015; 2016; 2017a; 2017b; 2017c; 2018; 2019; 2021; Heyn and Raja 2019) | Brooch, circular (Raja 2021) | Libation pitcher and incence bowl – often decorated (Raja 2015; 2016; 2017a; 2017b; 2017c; 2018; 2019; 2021; Heyn and Raja 2019) | – | – |
| 160–175 |  | Priestly hat, round, cylindrical, and flat-topped – often with a wreath and a central decoration (Raja 2015; 2016; 2017a; 2017c; 2016; 2018; 2019; 2021; Heyn and Raja 2019) | Shaved head (Raja 2015; 2016; 2017a; 2017b; 2017c; 2018) | – | – | No (Raja 2016; 2017a; 2017b; 2017c) | Tunic – occasionally embroidered or decorated (Raja 2015; 2016; 2017a; 2017b; 2017c; 2018; 2019; 2021; Heyn and Raja 2019)  Chlamys (Raja 2015; 2016; 2017a; 2017b; 2017c; 2018; 2019; 2021; Heyn and Raja 2019) | Brooch, circular (Raja 2021) | Libation pitcher and incence bowl – often decorated (Raja 2015; 2016; 2017a; 2017b; 2017c; 2018; 2019; 2021; Heyn and Raja 2019) | – | – |
| 175–220 |  | Priestly hat, round, cylindrical, and flat-topped – often with a wreath and a central decoration (Raja 2015; 2016; 2017a; 2017c; 2016; 2018; 2019; 2021; Heyn and Raja 2019) | Shaved head (Raja 2015; 2016; 2017a; 2017b; 2017c; 2018) | – | – | No (Raja 2016; 2017a; 2017b; 2017c) | Tunic – occasionally embroidered or decorated (Raja 2015; 2016; 2017a; 2017b; 2017c; 2018; 2019; 2021; Heyn and Raja 2019)  Chlamys (Raja 2015; 2016; 2017a; 2017b; 2017c; 2018; 2019; 2021; Heyn and Raja 2019) | Brooch, circular (Raja 2021) | Libation pitcher and incence bowl – often decorated (Raja 2015; 2016; 2017a; 2017b; 2017c; 2018; 2019; 2021; Heyn and Raja 2019) | – | – |
| 220–240 |  | Priestly hat, round, cylindrical, and flat-topped – often with a wreath and a central decoration (Raja 2015; 2016; 2017a; 2017c; 2016; 2018; 2019; 2021; Heyn and Raja 2019) | Shaved head (Raja 2015; 2016; 2017a; 2017b; 2017c; 2018) | – | – | No (Raja 2016; 2017a; 2017b; 2017c) | Tunic – occasionally embroidered or decorated (Raja 2015; 2016; 2017a; 2017b; 2017c; 2018; 2019; 2021; Heyn and Raja 2019)  Chlamys (Raja 2015; 2016; 2017a; 2017b; 2017c; 2018; 2019; 2021; Heyn and Raja 2019) | Brooch, circular (Raja 2021) | Libation pitcher and incence bowl – often decorated (Raja 2015; 2016; 2017a; 2017b; 2017c; 2018; 2019; 2021; Heyn and Raja 2019) | – | – |
| 240–272 |  | Priestly hat, round, cylindrical, and flat-topped – often with a wreath and a central decoration (Raja 2015; 2016; 2017a; 2017c; 2016; 2018; 2019; 2021; Heyn and Raja 2019) | Shaved head (Raja 2015; 2016; 2017a; 2017b; 2017c; 2018) | – | – | No (Raja 2016; 2017a; 2017b; 2017c) | Tunic – occasionally embroidered or decorated (Raja 2015; 2016; 2017a; 2017b; 2017c; 2018; 2019; 2021; Heyn and Raja 2019)  Chlamys (Raja 2015; 2016; 2017a; 2017b; 2017c; 2018; 2019; 2021; Heyn and Raja 2019) | Brooch, circular (Raja 2021) | Libation pitcher and incence bowl – often decorated (Raja 2015; 2016; 2017a; 2017b; 2017c; 2018; 2019; 2021; Heyn and Raja 2019) | – | – |

**Bibliography:**

Albertson, F. 2016. ‘Typology, Attribution, and Identity in Palmyran Funerary Portraiture’, in A. Kropp and R. Raja (eds), *The World of Palmyra,* Palmyrene Studies, 1 (Copenhagen: The Royal Danish Academy of Sciences and Letters), pp. 150–64.

Albertson, F. 2021. ‘The “Fringed” Mantle and its Relation to Gender in Palmyrene Funerary Sculpture’, in M. Heyn and R. Raja (eds), *Individualizing the Dead: Attributes in Palmyrene Funerary Sculpture*, Studies in Palmyrene Archaeology and History, 3 (Turnhout: Brepols), pp. 13–30.

Heyn, M. 2010. ‘Gesture and Identity in the Funerary Art of Palmyra’, *American Journal of Archaeology*, 114.4: 631–61.

Heyn, M. 2016. ‘Status and Stasis: Looking at Women in the Palmyrene Tomb’, in A. Kropp and R. Raja (eds), *The World of Palmyra,* Palmyrene Studies, 1 (Copenhagen: The Royal Danish Academy of Sciences and Letters), pp. 194–206.

Heyn, M. and R. Raja. 2019. ‘Male dress habit in Roman period Palmyra’, in M. Cifarelli (ed.), *Fashioned Selves: Dress and Identity in Antiquity* (Oxford: Oxbow Books), pp. 41–53.

Krag, S. 2017a. ‘Changing Identities, Changing Positions: Jewellery in Palmyrene Female Portraits’ in T. Long and A. H. Sørensen (eds), *Positions and Professions in Palmyra*, Palmyrene Studies, 2 (Copenhagen: The Royal Danish Academy of Sciences and Letters): pp. 36–51.

Krag, S. 2017b. ‘Palmyrene Funerary Female Portraits: Portrait Tradition and Change’, in M. Blömer and R. Raja (eds), *Funerary Portraiture in Greater Roman Syria*, Studies in Classical Archaeology, 6 (Turnhout: Brepols), pp. 111–31.

Krag, S. 2018. *Funerary Representations of Palmyrene Women: From the First Century BC to the Third Century AD*, Studies in Classical Archaeology, 3 (Turnhout: Brepols).

Krag, S. and R. Raja. 2016. ‘Representations of Women and Children in Palmyrene Funerary Loculus Reliefs, Loculus Stelae and Wall Paintings’, *Zeitschrift für Orient-Archäologie*, 9: 134–78.

Krag, S. and R. Raja. 2018. ‘Unveiling Female Hairstyles: Markers of Age, Social Rules, and Status in the Funerary Sculpture from Palmyra’, *Zeitschrift für Orient-Archäologie*, 11: 242–77.

Long, T. 2016. ‘Facing the Evidence: How to approach the portraits’, in A. Kropp and R. Raja (eds), *The World of Palmyra,* Palmyrene Studies, 1 (Copenhagen: The Royal Danish Academy of Sciences and Letters), pp. 135–49.

Thomsen, R. R. 2021. ‘Unlocking a Mystery? The Keys in Palmyrene Funerary Portraiture’, in M. Heyn and R. Raja (eds), *Individualizing the Dead: Attributes in Palmyrene Funerary Sculpture*, Studies in Palmyrene Archaeology and History, 3 (Turnhout: Brepols), pp. 51–62.

Raja, R. 2015. ‘Palmyrene Funerary Portraits in Context: Portrait Habit between Local Traditions and Imperial Trends’, in J. Fejfer, M. Moltesen, and A. Rathje (eds), *Traditions: Transmission of Culture in the Ancient World* (Copenhagen: Museum Tusculanum Press), pp. 329–61.

Raja, R. 2016. ‘Representations of Priests in Palmyra: Methodological Considerations on the Meaning of the Representation of Priesthood in Roman Period Palmyra’, *Religion in the Roman Empire,* 2,1: 125–46.

Raja, R. 2017a. ‘To be or not to be depicted as a priest in Palmyra: A matter of representational spheres and societal values’, in T. Long and A. H. Sørensen (eds), *Positions and Professions*, Palmyrene Studies, 2 (Copenhagen: The Royal Danish Academy of Sciences and Letters), pp. 115–30.

Raja, R. 2017b. ‘Representations of the so-called “former priests” in Palmyrene funerary art: A methodological contribution and commentary’, *Topoi*, 21: 51–81.

Raja, R. 2017c. ‘“You can leave your hat on”. Priestly representations from Palmyra: Between visual genre, religious importance and social status’, in R. Gordon, G. Peitridou, and J. Rüpke (eds), *Beyond Priesthood: Religious Entrepreneurs and Innovators in the Roman Empire* (Berlin: De Gruyter), pp. 417–42.

Raja, R. 2018. ‘The matter of the Palmyrene “modius”. Remarks on the history of research into the terminology of the Palmyrene priestly hat’, in R. Raja and J. Rüpke (eds), *Religious Terminology*, Religion in the Roman Empire 2:4 (Tübingen: Mohr Siebeck), pp. 237–59.

Raja, R. 2019. ‘It stays in the family: Palmyrene priestly representations and their constellations’, in S. Krag and R. Raja (eds), *Women, children and the family in Palmyra*, Palmyrene Studies, 3 (Copenhagen: The Royal Danish Academy of Sciences and Letters), pp. 95–156.

Raja R. 2021. ‘Managing the Middle Ground: Priests in Palmyra and their iconographies’, in J. Hoffmann-Salz (ed.), *The Middle East as Middle Ground? Cultural Interaction in the Ancient Middle East Revisited* (Vienna: Holzhausen), pp. 129–46.

Raja, R. and J.-B. Yon. Forthcoming. ‘Palmyrene funerary sculptural representations with Greek, Latin and bilingual inscriptions’, *Zeitschrift für Orient-Archäologie*.
