# Supplementary material for: Three hundred years of Palmyrene history. Unlocking archaeological data for studying past societal transformations
Source: PLoS One. 2021 Nov 3;16(11):e0256081. doi: 10.1371/journal.pone.0256081 (PMC8565770; doi:10.1371/journal.pone.0256081)
Supplement: S1 File — (DOCX) [file pone.0256081.s010.docx]

The Supporting Information files can be downloaded through this link:

<https://www.dropbox.com/sh/dsxfjdrv1hix371/AAA08g-5CSg9WxziUVq7Vqf3a?dl=0>
